# Supplementary material for: Discovery of DNA aptamers targeting SARS-CoV-2 nucleocapsid protein and protein-binding epitopes for label-free COVID-19 diagnostics
Source: Mol Ther Nucleic Acids. 2023 Feb 14;31:731–43. doi: 10.1016/j.omtn.2023.02.010 (PMC9927813; doi:10.1016/j.omtn.2023.02.010)
Supplement: Document S1. Figures S1–S7 and Tables S1–S3 [file mmc1.pdf]

**OMTN, Volume *31***

## Supplemental information

# Discovery of DNA aptamers targeting SARS-CoV-2 nucleocapsid protein and protein-binding epitopes for label-free COVID-19 diagnostics

**Suttinee Poolsup, Emil Zaripov, Nico Hüttmann, Zoran Minic, Polina V. Artyushenko, Irina A. Shchugoreva, Felix N. Tomilin, Anna S. Kichkailo, and Maxim V. Berezovski**

**SARS-CoV-2 Protein expression**

DH5α strain with a plasmid encoding for N protein was received from Rick Tarleton (Addgene, Watertown, MA). An LB agar plate containing 100 µg/mL kanamycin (Sigma-Aldrich, St. Louis, MO) was inoculated with the culture and incubated at 37°C for 24 hours. A single colony was picked and inoculated into 5 mL LB with 100 µg/mL kanamycin, which was incubated at 300 rpm and 37°C overnight. The plasmid was extracted using a miniprep kit (Qiagen, Germantown, MD), and 10 ng of the plasmid was used to transform into competent cells (DE3 strain) (Thermo Fisher Scientific, Waltham, MA) according to the manufacturer's protocol. The transformed competent cells were cultured in the same conditions as DH5α and were frozen in 50% glycerol/LB. The frozen cells were inoculated into 200 mL LB with 100 µg/mL kanamycin. The culture was incubated at 37°C on a magnetic stir plate at 300 rpm until a OD600 reached 1. The culture was then supplied with 1mM IPTG (Sigma-Aldrich, St. Louis, MO) and further incubated for 3 hours. Cells were spun down at 4,000 g for 30 minutes and resuspended in T300 buffer (30mM triethanolamine, 300 mM NaCl, pH 7.8). 1mg/mL of lysozyme and 1mM PMSF (Sigma-Aldrich, St. Louis, MO) were added before sonication for 30 minutes on ice. The lysate was centrifugated at 21,000 g for 40 minutes, and pellets were resuspended in T300 supplied with 8 M urea (Sigma-Aldrich, St. Louis, MO). The solubilized pellets were then filtered through a 0.2-µm nylon membrane. The C 10/10 chromatography column (Cytiva, Marlborough, MA) was packed with 7.85 mL of Ni-NTA agarose beads (Thermo Fisher Scientific, Waltham, MA). Packed media was equilibrated with T300 buffer containing 8 M urea for 4 column volumes. 10 mL of solubilized sample was injected into the column, which was then washed with 4 more column volumes of the same buffer containing urea. The buffer was slowly replaced with T300 by applying gradient for 8 column volumes to gradually remove urea and refold protein on the column. Column was then

washed with T300 with 30 mM imidazole for 4 column volumes. The protein was then eluted with 8 column volumes of elution buffer (T300 and 300mM imidazole) and concentrated on a 10 kDa filter (Sigma-Aldrich, St. Louis, MO). All chromatography steps were performed using a flow rate of 3 mL/min. In the next step, gel filtration chromatography was performed using a XK 16/70 column (Cytiva, Marlborough, MA) which was packed with 140 mL of Sephacryl S-100 HR resin (Cytiva, Marlborough, MA). The column was washed with 2 volumes of gel filtration buffer (50 mM HEPES, 50 mM NaCl and 10 mM $MgCl_2$, pH 7.5). 1 mL of concentrated sample was injected and separated at a flow rate of 0.5 mL/min. A280 was monitored during the run to collect fractions of N protein. The collected protein was then concentrated on a 10 kDa filter to the final volume of 1 mL and stored at -20 °C until further use.

**Table S1**. Full-length sequences (80 nt) of all selected aptamer candidates were obtained from NGS data enrichment. The primer-binding regions are written in bold.

| Aptamer | Aptamer sequence |
|---|---|
| NSP1 | **CTCCTCTGACTGTAACCACG**GCGCAAGCCGGGGTGTACGTGTT ATACGTGCGTGTATCGA**GCATAGGTAGTCCAGAAGCC** |
| NSP2 | **CTCCTCTGACTGTAACCACG**TATTGCGTTCCAGTCCCTATGACC AACGTCACAATAAGTC**GCATAGGTAGTCCAGAAGCC** |
| NSP3 | **CTCCTCTGACTGTAACCACG**CAGCGTCACGTGTTGTTCCCCATT GTACTGATTCGTCGTG**GCATAGGTAGTCCAGAAGCC** |
| NSP4 | **CTCCTCTGACTGTAACCACG**CGTTGAGCGTGTTGTCCCTACAT GCAATAGACCCTCCTTC**GCATAGGTAGTCCAGAAGCC** |
| NSP5 | **CTCCTCTGACTGTAACCACG**ATTCATGCGCCAATAGTGGTTTG GAAATGTCTCCCCATAC**GCATAGGTAGTCCAGAAGCC** |
| NSP6 | **CTCCTCTGACTGTAACCACG**GCATAGGTAGTCAAGAAGCCATC TCCTATGACTGTAACCACG**GCATAGGTAGTCCAGAAGCC** |
| NSP7 | **CTCCTCTGACTGTAACCACG**TGCTAGATTACGTAACTGGTTGC TACGGGTTTCATCCTCG**GCATAGGTAGTCCAGAAGCC** |
| NSP8 | **CTCCTCTGACTGTAACCACG**AGGATTGCGCACATACGGTTGCT CACCATTCTACTGTCCT**GCATAGGTAGTCCAGAAGCC** |
| NSP9 | **CTCCTCTGACTGTAACCACG**CTTATGCACCAGGGGCCCGGTTC GGGCAGGCGCAAACACG**GCATAGGTAGTCCAGAAGCC** |
| NSP10 | **CTCCTCTGACTGTAACCACG**GCATAGGTAGTCAAGAAGCCATC TCATCTGACTGTAACCACG**GCATAGGTAGTCCAGAAGCC** |
| NSP11 | **CTCCTCTGACTGTAACCACG**GCGCACCCTGCGACGTCCTCATG CCAGGCTTCAGCCTCAA**GCATAGGTAGTCCAGAAGCC** |
| NSP12 | **CTCCTCTGACTGTAACCACG**TGGTTCGCCCAGTTCCGTTCAAT ACACTCAACCCTGCACG**GCATAGGTAGTCCAGAAGCC** |
| NSP13 | **CTCCTCTGACTGTAACCACG**TATTGCGTTCCATTCCCTATGACC AACGTCACAATAAGTC**GCATAGGTAGTCCAGAAGCC** |
| NSP14 | **CTCCTCTGACTGTAACCACG**GGCTTGGTTTCGGGATCTGCACC CCTGAAACATTTCTCAG**GCATAGGTAGTCCAGAAGCC** |
| NSP15 | **CTCCTCTGACTGTAACCACG**CTCGTCCAGCGGCTGCGTGTTTC ACCGTGCATTGTTGATA**GCATAGGTAGTCCAGAAGCC** |
| NSP16 | **CTCCTCTGACTGTAACCACG**TTCAAGAGCGGTTCGTAATGTTA ATTGACTGATCCCTACC**GCATAGGTAGTCCAGAAGCC** |

**Table S2**. The truncated aptamer sequences and their binding parameters interacting with His-tagged N protein immobilized on the Ni-NTA biosensors.

| Aptamer | Aptamer sequence | $K_D$ (nM) | $k_a$ (1/Ms) | $k_d$ (1/s) |
|---|---|---|---|---|
| tNSP5 (46nt) | ATTCATGCGCCAATAGTGGTTTGGAA ATGTCTCCCCATACGCATAG | 12.6±2.56 | $8.25x10^5$ | $1.07x10^{-2}$ |
| tNSP9 (60nt) | TGTAACCACGTGCTAGATTACGTAAC TGGTTGCTACGGGTTTCATCCTCGGCA TAGGTA | 22.2±1.87 | $4.77x10^5$ | $1.06x10^{-2}$ |
| tNSP10 (47nt) | CTTATGCACCAGGGGCCCGGTTCGGG CAGGCGCAAACACGGCATAGG | 8.60±1.20 | $7.44x10^5$ | $6.39x10^{-3}$ |
| tNSP12 (60nt) | TGTAACCACGTGGTTCGCCCAGTTCCG TTCAATACACTCAACCCTGCACGGCA TAGGTA | 6.05±2.41 | $6.14x10^5$ | $3.99x10^{-3}$ |
| A48 (58 nt) | GCTGGATGTCGCTTACGACAATATTCC TTAGGGGCACCGCTACATTGACACAT CCAGC | 6.46±1.04 | $7.82x10^5$ | $5.05x10^{-3}$ |
| A58 (58 nt) | GCTGGATGTCACCGGATTGTCGGACA TCGGATTGTCTGAGTCATATGACACAT CCAGC | 3.09±0.95 | $3.58x10^5$ | $1.08 x10^{-3}$ |

**Table S3.** Hydrogen bonds in the N protein-tNSP3 complexes. The number in parentheses indicates the conformer number. Amino acids of AA10 peptide are in bold.

| N protein/NSP3 (1) | | N protein/NSP3 (2) | | N protein/NSP3 (1) | | N protein/NSP3 (2) | |
|---|---|---|---|---|---|---|---|
| **Binding site AA21** | | **Binding site AA21** | | **Binding site AA10** | | **Binding site AA10** | |
| Residue | Nucleotide | Residue | Nucleotide | Residue | Nucleotide | Residue | Nucleotide |
| GLN 9 | G 35 | GLN 9 | G 35 | LYS 338 | C 4 | ASN 126 | T 23 |
| ARG 10 | G 35 | ARG 10 | G 35 | LYS 361 | C 37 | LYS 127 | T 23 |
| ARG 14 | C 34 | ASN 29 | C 1 | GLU 367 | G 30 | ARG 276 | T 23 |
| ASN 29 | T 6 | **ASN 77** | T 32 | **ARG 385** | T 17 | ARG 293 | C 19 |
| ASN 29 | A 2 | **SER 79** | T 23 | **GLN 384** | G 15 | LYS 338 | C 4 |
| GLU 31 | C 1 | THR 14 | C 21 | **GLN 384** | C 18 | LYS 361 | C 37 |
| GLU 31 | A 2 | LYS 143 | C 19 | GLN 386 | T 14 | GLU 367 | G 30 |
| SER 37 | C 1 | LYS 143 | C 20 | LYS 387 | T 16 | LYS 373 | T 29 |
| **ASN 77** | T 32 | GLN 160 | G 25 | LYS 387 | T 14 | LYS 375 | T 33 |
| **SER 79** | A 22 | ARG 189 | A 27 | GLN 408 | C 4 | **GLN 384** | G 15 |
| **SER 79** | T 23 | SER 193 | T 29 | GLN 408 | T 6 | **GLN 384** | C 18 |
| LYS 143 | C 20 | LYS 237 | G 3 | | | **GLN 384** | C 19 |
| GLN 160 | G 25 | | | | | **ARG 385** | T 17 |
| GLN 163 | T 24 | | | | | **ARG 385** | C 18 |
| SER 193 | T 29 | | | | | GLN 386 | T 14 |
| SER 201 | T 29 | | | | | GLN 386 | T 16 |
| ARG 203 | G 30 | | | | | LYS 387 | T 16 |
| ARG 203 | T 29 | | | | | GLN 408 | C 4 |
| LYS 237 | G 3 | | | | | | |
| LYS 256 | A 2 | | | | | | |

**A**

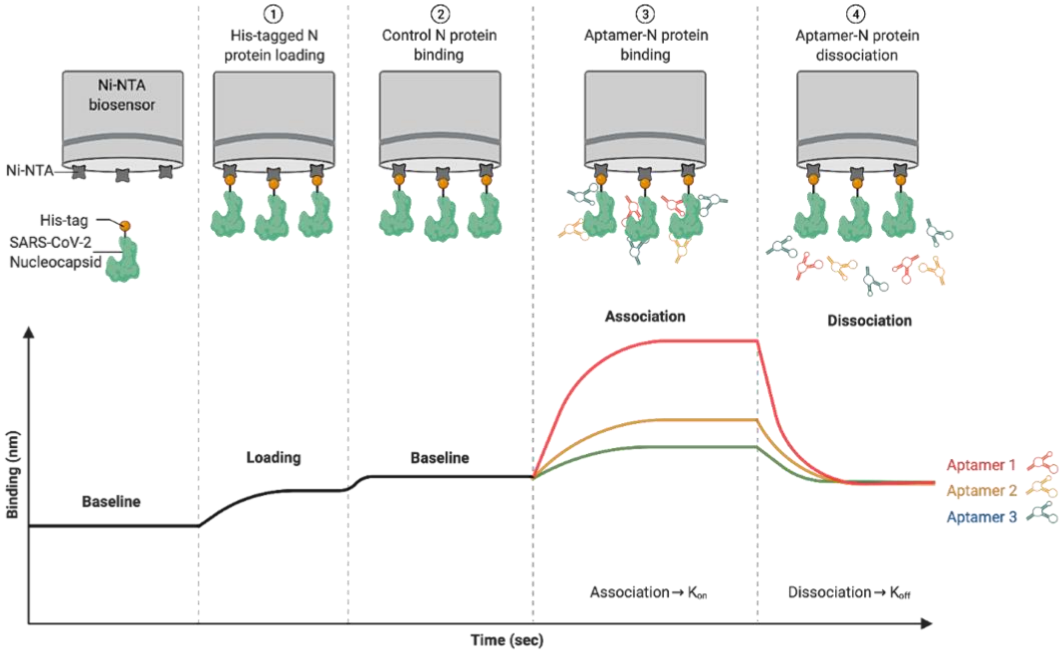


**B**

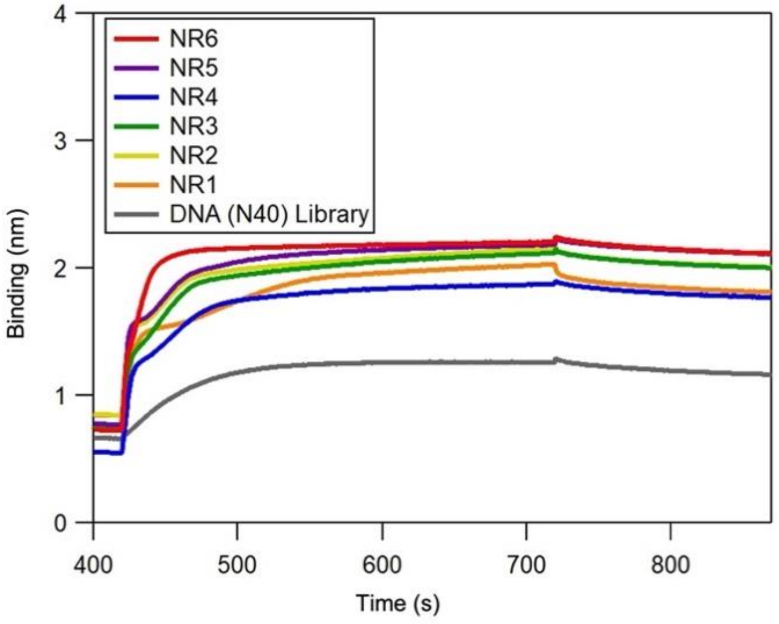


**Figure S1**. **BLI affinity tests.** (A) Schematic BLI workflow of aptamers binding to N protein. (B) BLI sensorgrams showing the association and dissociation curves of the six enriched pools obtained from the aptamer selection.

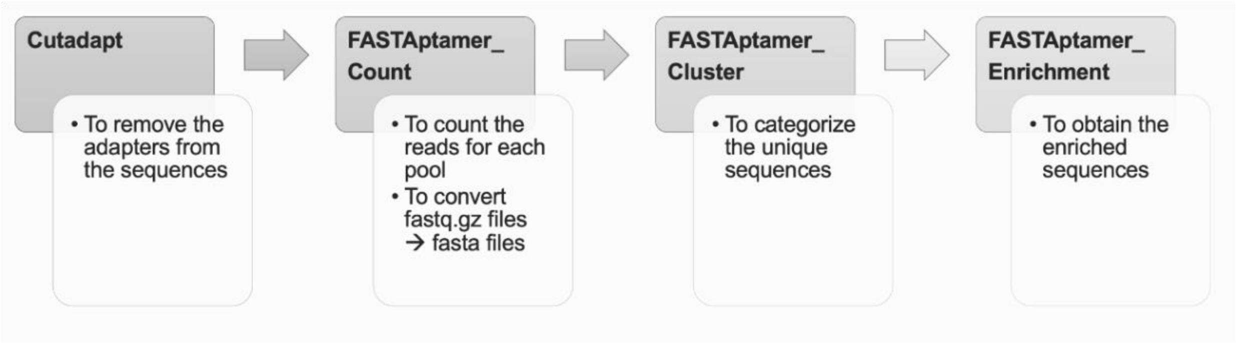


**Figure S2. Schematic NGS data analysis.** The data processing workflow to retrieve the aptamer sequences from NGS raw data.[36]

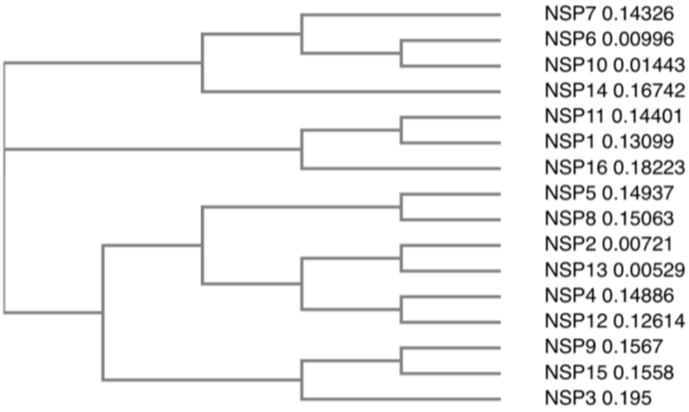


**Figure S3**. **Phylogenetic tree of DNA aptamers.** The diagram obtained from Clustal Omega represents the three main groups of sixteen enriched aptamer sequences.

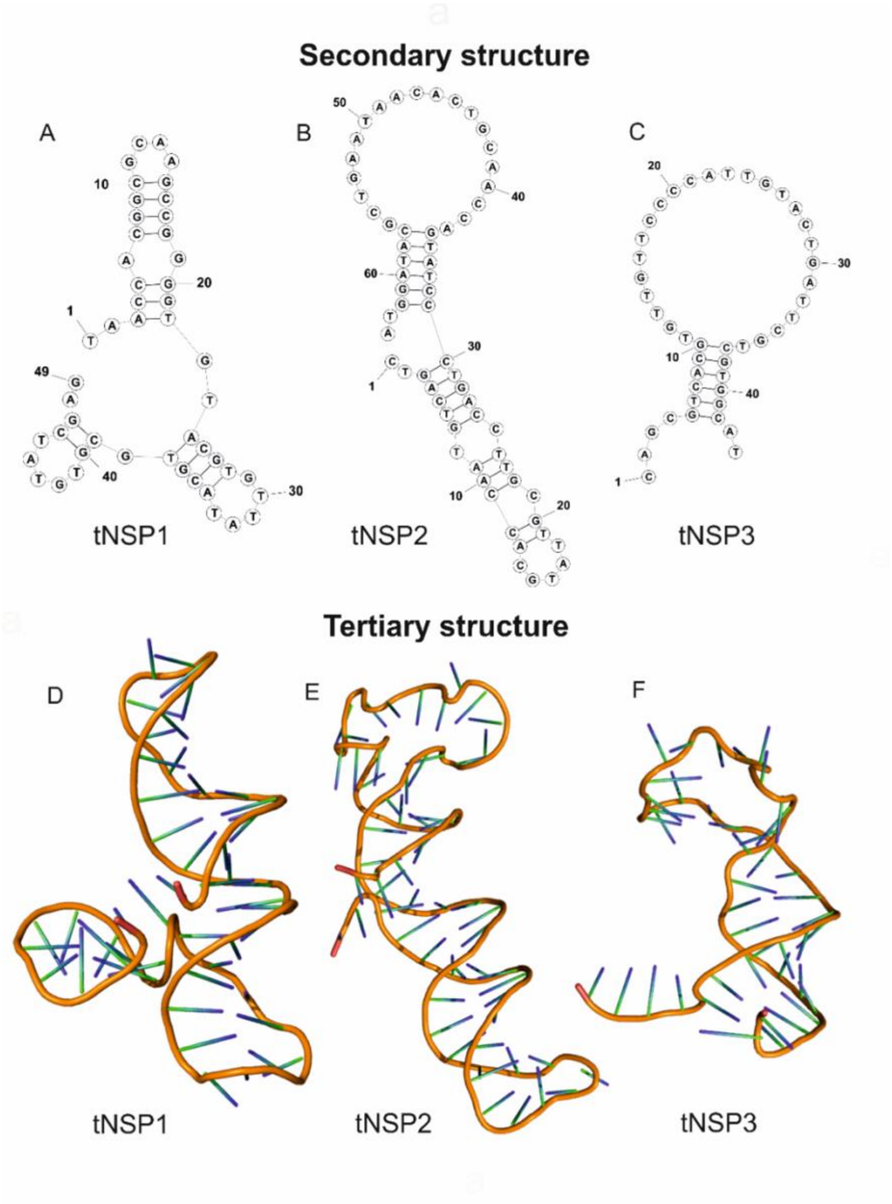


**Figure S4. The structural prediction of truncated aptamers.** Secondary structures (A-C) of tNSP1, tNSP2, and tNSP3 aptamers predicted by the mFold web server.[68] Tertiary structures (D-E) obtained from MD simulations (200 ns).

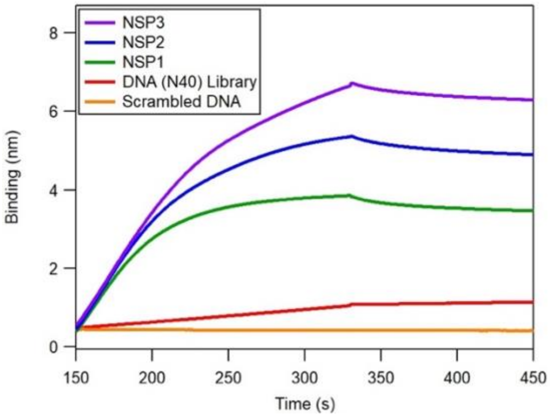


**Figure S5**. **Binding evaluation on BLI.** Binding affinity tests of NSP1, NSP2, and NSP3 aptamers with the N protein.  The initial DNA N40 library and scrambled DNA were used as controls.

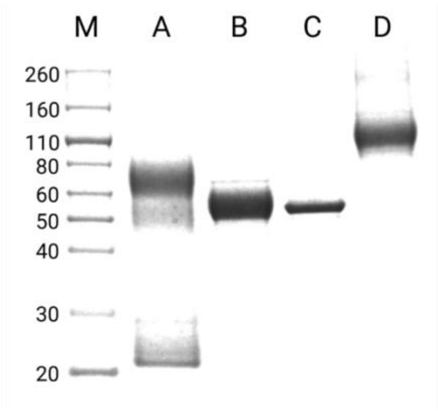


**Figure S6. SDS-PAGE gel image with four proteins.** M – molecular marker; A – nucleocapsid of MERS-CoV; B – nucleocapsid of SARS-CoV-2 obtained from ACROBiosystems; C – nucleocapsid of SARS-CoV-2 expressed in BL-21 *E. coli*. D. S1 subunit of the spike protein of SARS-CoV-2 obtained from ACROBiosystems.

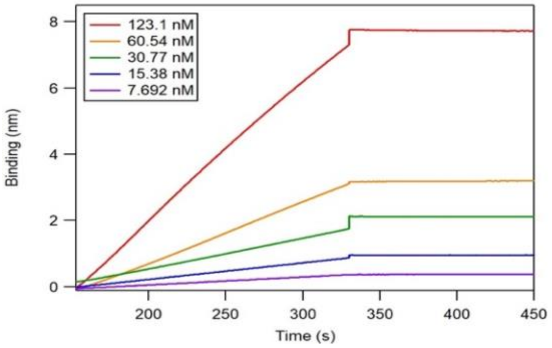


**Figure S7**. **BLI sensorgrams of tNSP3 aptamer.** The binding assay determining $K_D$ of tNSP3 aptamer binding to our in-house expressed SARS-CoV-2 nucleocapsid protein at 7.69, 15.38, 30.76, 61.52, and 123.04 nM.
