# Supplementary material for: Discovery of DNA aptamers targeting SARS-CoV-2 nucleocapsid protein and protein-binding epitopes for label-free COVID-19 diagnostics
Source: Mol Ther Nucleic Acids. 2023 Feb 14;31:731–43. doi: 10.1016/j.omtn.2023.02.010 (PMC9927813; doi:10.1016/j.omtn.2023.02.010)
Supplement: Document S2. Article plus supplemental information [file mmc2.pdf]

Molecular Therapy
Nucleic Acids
Original Article

Discovery of DNA aptamers targeting SARS-CoV-2 nucleocapsid protein and protein-binding epitopes for label-free COVID-19 diagnostics

Suttinee Poolsup,[1] Emil Zaripov,[1] Nico Hüttmann,[2] Zoran Minic,[2] Polina V. Artyushenko,[3,4,5] Irina A. Shchugoreva,[3,4,5] Felix N. Tomilin,[5,6] Anna S. Kichkailo,[3,4] and Maxim V. Berezovski[1,2]

[1]Department of Chemistry and Biomolecular Sciences, University of Ottawa, Ottawa, ON K1N 6N5, Canada; [2]John L. Holmes Mass Spectrometry Facility, Faculty of Science, University of Ottawa, Ottawa, ON K1N 6N5, Canada; [3]Laboratory for Digital Controlled Drugs and Theranostics, Federal Research Center "Krasnoyarsk Science Center SB RAS", Krasnoyarsk 660036, Russia; [4]Prof. V.F. Voino-Yasenetsky Krasnoyarsk State Medical University, Krasnoyarsk 660022, Russia; [5]Department of Chemistry, Siberian Federal University, Krasnoyarsk 660041, Russia; [6]Laboratory of Physics of Magnetic Phenomena, Kirensky Institute of Physics, Krasnoyarsk 660036, Russia

The spread of COVID-19 has affected billions of people across the globe, and the diagnosis of viral infection still needs improvement. Because of high immunogenicity and abundant expression during viral infection, SARS-CoV-2 nucleocapsid (N) protein could be an important diagnostic marker. This study aimed to develop a label-free optical aptasensor fabricated with a novel single-stranded DNA aptamer to detect the N protein. The N-binding aptamers selected using asymmetric-emulsion PCR-SELEX and their binding affinity and cross-reactivity were characterized by biolayer interferometry. The tNSP3 aptamer (44 nt) was identified to bind the N protein of wild type and Delta and Omicron variants with high affinity (K_D in the range of 0.6–3.5 nM). Utilizing tNSP3 to detect the N protein spiked in human saliva evinced the potential of this aptamer with a limit of detection of 4.5 nM. Mass spectrometry analysis was performed along with molecular dynamics simulation to obtain an insight into how tNSP3 binds to the N protein. The identified epitope peptides are localized within the RNA-binding domain and C terminus of the N protein. Hence, we confirmed the performance of this aptamer as an analytical tool for COVID-19 diagnosis.

INTRODUCTION

With the rapid spread of a novel coronavirus identified as severe acute respiratory syndrome coronavirus 2 (SARS-CoV-2), the number of COVID-19 cases dramatically increased and later became a global pandemic.[1] Thus far, approximately 539 million cases and more than 6.3 million deaths have been confirmed worldwide.[2] To decrease the mortality rate, it is necessary to develop robust and fast diagnostic tools that reflect the early stages of viral infection. According to various manifestations of COVID-19, including asymptomatic infections, a molecular technique such as RT-PCR is primarily used for COVID-19 diagnosis.[3–5] Among four structural proteins, including spike surface glycoprotein, envelope protein, membrane protein, and nucleocapsid (N) protein,[6] the genes encoding spike, envelope, and N have been targeted for PCR testing.[7–9] Because of the complex pre-treatment, processing time, and the cost of RT-PCR,[10,11] COVID-19 antibody tests, also known as serological tests, have been the only alternative to nucleic acid testing officially approved by the US Food and Drug Administration.[12] The serological test can rapidly identify infected people and nearby contacts.[13] However, serological-based assays, e.g., ELISA and lateral flow immunoassay, essentially rely on the function of antibodies such as immunoglobulin G (IgG)/IgM implemented on the sensing materials,[14–16] which can present a high risk of false-negative results caused by inadequate antibodies in clinical samples and inconsistent immobilization of antigen-coated supporting materials, while the varying range of viral load over the infection period can cause inaccuracy of antigen detection.[17] Like antibodies, aptamers selectively bind and recognize target antigens or specific proteins; nonetheless, aptamers are smaller, chemically synthesized, and more stable with ease of chemical modification, and have less batch-in-batch variation compared with antibodies.[18,19] Aptamers are short nucleic acids and peptides produced *in vitro* through the systematic evolution of ligands by exponential enrichment (SELEX).[20,21] Remarkably, the applications of aptamers for viral diagnostics and therapeutics have been drastically increasing in the past decade since the emergence of the SARS coronavirus in 2002.[22,23] While most studies have been paying attention to the receptor-binding domain and spike protein of SARS-CoV-2,[24–26] SARS-CoV-2 N, an RNA-binding protein for viral genome encapsulation, is abundantly expressed in infected cells with very low genetic variation over time compared with the spike protein.[27,28] In this regard, the N protein could be used as a potential biomarker for early diagnosis.[29] In the early days of the global

Received 3 August 2022; accepted 10 February 2023;
https://doi.org/10.1016/j.omtn.2023.02.010.

Correspondence: Maxim V. Berezovski, Department of Chemistry and Biomolecular Sciences, University of Ottawa, Ottawa, ON K1N 6N5, Canada.
E-mail: maxim.berezovski@uottawa.ca

This is an open access article under the CC BY license (http://creativecommons.org/licenses/by/4.0/).

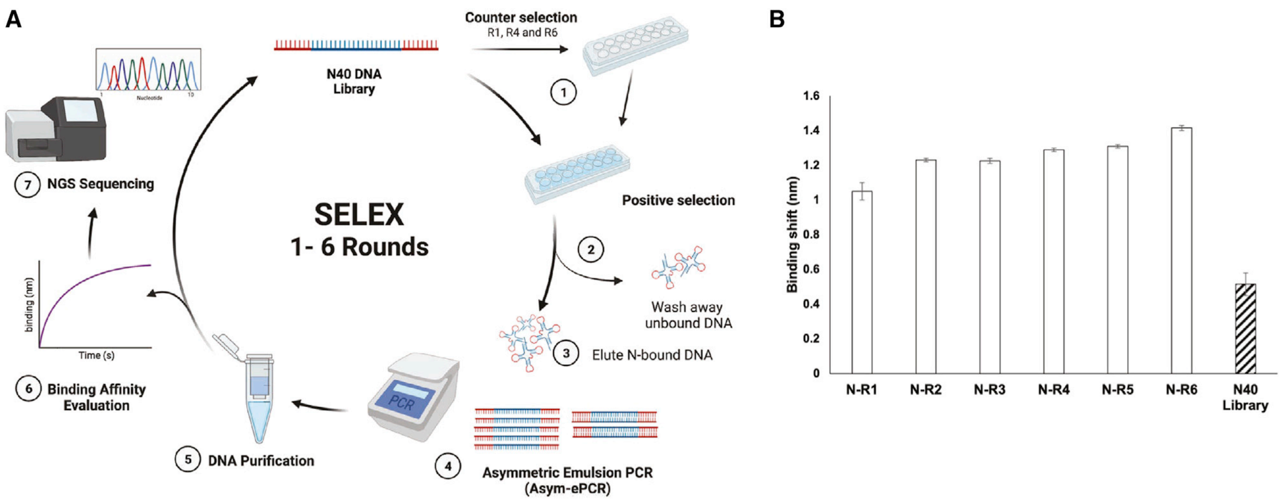

Figure 1. Asymmetric-emulsion PCR-SELEX

(A) Schematic representation of asymmetric-emulsion PCR (ePCR) SELEX. (B) N protein binding evaluation of six enriched DNA pools obtained from asymmetric-ePCR SELEX compared with an initial DNA N40 library using BLI. Error bars represent means ± SD. See also Figure S1B.

pandemic, Zhang and co-workers[30] reported the DNA aptamers targeting SARS-CoV-2 N protein and implemented the selected aptamers with an anti-N antibody for N protein detection. Thereafter, several studies used the reported aptamer sequences to implement various types of biosensors aiming to improve the sensitivity and accuracy in detecting SARS-CoV-2 N.[31–33] These studies are still ongoing but carry the disadvantages of requiring multiple steps of sample preparation and a lack of understanding of molecular interaction between the N protein and aptamers. In this study, we selected novel single-stranded DNA (ssDNA) aptamers using asymmetric-emulsion PCR-SELEX to target SARS-CoV-2 N protein strategies and sequences different from those of previous studies. The aptamer sequence, possessing the highest affinity to the N protein, was fabricated on biolayer interferometry (BLI) through the biotin-streptavidin interaction without additional labels. This label-free aptamer-based BLI was successfully able to detect the SARS-CoV-2 N protein of the wild type as well as Delta and Omicron variants. Furthermore, we studied the cross-reactivity of the selected aptamers to other SARS-CoV-2 and MERS proteins. In addition to the discovery of DNA aptamers targeting the N protein, the technology of aptamer-facilitated biomarker discovery (AptaBiD)[34] was amenable to identification of the specific binding epitope on the SARS-CoV-2 N protein using our selected aptamer. The aptamer-binding digested peptides were processed through a proteomics approach, and the interaction of the binding epitope and the aptamer was additionally assessed by molecular dynamics (MD) simulation. This study can be used not only to distinguish between SARS-CoV-2 positive and negative clinical samples but also to identify aptamer-protein binding motifs that may also be applied to therapeutic purposes in the future. Hence, the use of this aptamer-based BLI has highlighted the potential of our discovered aptamer as a promising candidate for COVID-19 diagnostics.

RESULTS

Selection of DNA aptamers to SARS-CoV-2 nucleocapsid protein

The recombinant N protein was used as a target for ssDNA aptamer selection using the SELEX procedure shown in Figure 1A. The DNA library, with a 40-nt random region flanked with 20-nt primer-binding sequences at 5′ and 3′ ends, was incubated with the N protein immobilized on nickel-nitrilotriacetic acid (Ni-NTA) HisSorb strips. As thoroughly described in materials and methods, the unbound DNA sequences were washed out while the N-protein-binding DNA pool was further amplified by asymmetric-emulsion PCR and purified with a PCR cleanup kit to generate an ssDNA pool subsequently subjected to the next rounds of selection. To eliminate the non-specific sequences, the counter-selection took place in the first, fourth, and sixth rounds of selection before starting the positive selection. After each round of selection, one additional wash was added to remove the weak-binding ssDNA sequences and increase the stringency of the selection, while in addition the number of PCR cycles was optimized to achieve maximum amplification efficiency.

To assess the presence of the desired ssDNA sequences with 80-nt length in each enriched pool from every round of selection, gel electrophoresis was carried out, whereby the results showed two bands of ssDNA as a major product and double-stranded DNA as a minor product located at 40 bp and 80 bp, respectively. After six selection rounds, the enriched pools were subjected to preliminary binding affinity tests using BLI. The binding and dissociation of the target protein and the analytes on the biosensor tips affect the reflected light's interference pattern, indicating the change in molecular thickness on the tip surface.[35] This phenomenon can be reported as a change in wavelength (nm) over time, as in the BLI sensorgrams shown in Figure S1A. All six enriched pools showed relatively higher binding

shifts (Figure 1B) with a 2-fold increase of the association curves compared with the initial N40 DNA library after 300-s partitioning with N protein on the Ni-NTA biosensor tips (Figure S1B). Therefore. all enriched pools were sequenced and analyzed using next-generation sequencing (NGS).

The NGS results for all six pools were processed using the FASTAptamer toolkit (Figure S2).[36] Sequences were counted and clustered into families based on the neighboring distance, which were further subjected to the enrichment tool to calculate the fold enrichment of individual sequences across six pools. The retrieved sequences were chosen on the basis of three criteria, namely (1) the total length of 80–82 nucleotides containing 40–42 nt random region flanked with forward and reverse primers, (2) the identical sequences enriched in more than one pool, and (3) the sequence composition without primer-dimer formation in the random region. While three enriched sequences named NSP1, NSP2, and NSP3 were obtained from the cluster-enrichment tool, the count-enrichment tool resulted in 16 unique sequences (Table S1) based on these criteria, including the NSP1, NSP2, and NSP3 aptamer sequences. These 16 unique sequences were then aligned to analyze the differentiation and similarity using Clustal Omega.[37] Relative to the construction of the N40 DNA library with a 40-nt randomized region, the insertion of two nucleotides was observed in two sequences named NSP6 and NSP10. Phylogenetic tree analysis based on primary sequence homology categorized the 16 aptamer sequences into three groups (Figure S3). Among the 16 unique sequences, the truncated structures of seven aptamers (tNSP1, tNSP2, tNSP3, tNSP5, tNSP9, tNSP10, and tNSP12) representing each subgroup were chosen for further validation of their affinity binding to the N protein. Moreover, the secondary structures were predicted using an RNAstructure web server for designing the truncated motifs of aptamers.[38] Based on the observation of the predicted structures, some additional nucleotides at the 5′ and 3′ primer-binding regions remained to conserve the stem-loop structures. This results in different nucleotide lengths of the selected aptamers in the range of 47–60 nt (Table S2). According to the highly enriched characteristic of the NSP1, NSP2, and NSP3 aptamers, the apparent dissociation constant (K_D) values of the full-length structures of these three aptamers were determined using BLI.

K_D determination for the selected aptamers

To study the real-time binding assay on the BLI instrument (named Octet N1), serial dilutions of NSP1, NSP2, NSP3, and their truncated structures were prepared in the assay buffer with five different concentrations of aptamer from 200 nM to 12.5 nM. The reference assay was prepared in the absence of an aptamer. The his-tagged N protein was loaded on an Ni-NTA biosensor, after which the sensor tip was dipped into the aptamer solution as an analyte for association and dissociation steps. The results demonstrated the binding of full-length NSP1, NSP2, and NSP3 aptamers to the N protein with K_D values of 7.86 ± 1.17, 4.38 ± 1.21, and 8.60 ± 1.06 nM, respectively. The K_D values of truncated structures (Figure S4) of these three aptamers named tNSP1, tNSP2, and tNSP3 were also determined to study the impact of primer-binding regions on the target recognition, giving K_D values of 4.53 ± 0.96, 3.04 ± 1.53, and 5.77 ± 1.17 nM, respectively. These results indicate that the primer-binding regions did not significantly affect the binding affinity. Also, the other four aptamers (tNSP5, tNSP9, tNSP10, and tNSP12) were truncated, and their K_D values ranged from 6 nM to 22 nM (Table S2). In addition to the binding assay of the selected aptamers, the initial N40 DNA library and the scrambled DNA with 80 nt were tested as controls. The DNA library showed low binding affinity and a low BLI shift (about 0.5 nm) to the N protein and insignificant binding behavior of the scrambled DNA (Figure S5). As positive controls, the binding affinity of two previously published aptamers (A48 and A58 with reported K_D values of 0.49 ± 0.95 and 0.70 ± 0.06 nM, respectively)[30] were measured on Octet N1, and their K_D values were 6.46 ± 1.04 and 3.02 ± 0.95 nM, respectively. According to our study, Mg^{2+} and Ca^{2+} ions played a crucial role in maintaining the binding affinity between DNA aptamers and the protein compared with the Mg/Ca-omitted buffer condition so that these divalent ions were always added into the binding and assay buffers. Owing to the high affinity of the three truncated aptamers (tNSP1, tNSP2, and tNSP3), they were further used for subsequent studies.

Binding evaluation of aptamers to SARS-CoV-2 N protein

To establish the detection of the N protein using aptamers, affinity studies were also performed by individually immobilizing the biotinylated tNSP1, tNSP2, and tNSP3 aptamers on a streptavidin biosensor, SARS-CoV-2 N protein was spiked in PBS with Tween 20 (PBST) buffer with varied protein concentrations of 7.69, 15.38, 30.76, 61.52, and 123.04 nM, and the control assay was prepared in the absence of the protein. There was no significant difference in K_D values obtained from attaching either the truncated aptamers or the N protein on the biosensors. The analysis setup with the aptamer-loaded sensor (Figure 2A) yielded a better curve fit (R^2 = 0.999) with the use of the default binding model (1:1) of the BLI software, which refers to the higher accuracy of the calculated K_D. Kinetic binding of these three aptamers (Figures 2B–2D) showed a potential capability of N protein detection in PBST buffer with K_D values of 4.83 ± 1.09, 4.51 ± 1.50, and 2.91 ± 1.02 nM for tNSP1, tNSP2, and tNSP3, respectively (Table 1).

Assessment of aptamer selectivity for SARS-CoV-2 nucleocapsid

To verify the selectivity of the tNSP1, tNSP2, and tNSP3 aptamers to the SARS-CoV-2 N protein, their binding affinity to other viral proteins was tested, including SARS-CoV-2 S1 protein and the MERS coronavirus nucleocapsid (MERS N). The purity of each protein was visualized on SDS-PAGE (Figure S6), and each protein displayed a single band representing high homogeneity. All three aptamers exhibited strong binding to SARS-CoV-2 N protein (Figures 3A–3C) with no significant cross-reactive binding to the SARS-CoV-2 S1. Some binding (about 50%) was observed to the N protein of MERS coronavirus, highlighting the higher affinity of these aptamers to SARS-CoV-2. Notably, these three truncated aptamers did not exhibit binding to hexahistidine peptide used as a tag on the recombinant proteins in this study. Considering the lowest K_D and highest selectivity to the N protein, the tNSP3 aptamer was chosen for detecting

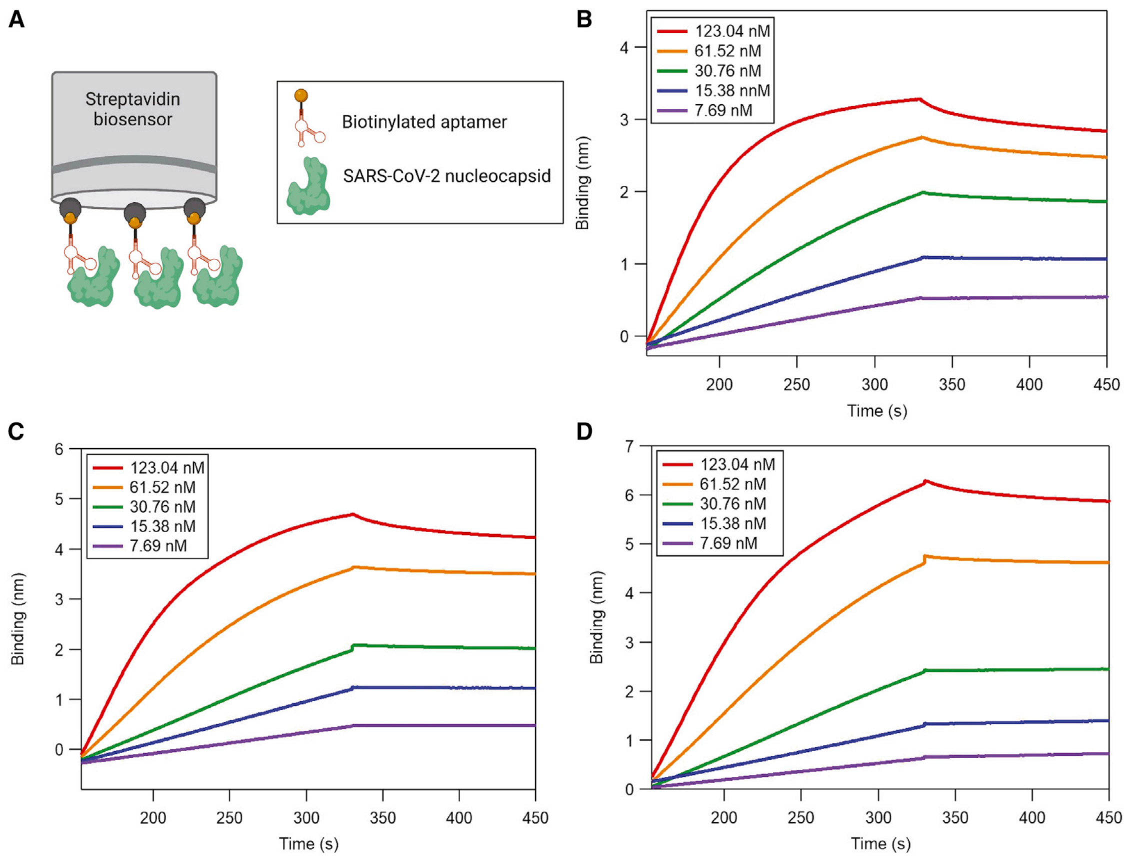

Figure 2. Real-time detection of aptamer binding to SARS-CoV-2 N protein

(A) Scheme of an aptamer-based BLI detection setup. The biotinylated aptamer was immobilized on a streptavidin biosensor and incubated with SARS-CoV-2 N protein. The binding signals were detected in real time as the association (180 s) and dissociation (120 s) of aptamers and the N protein. (B–D) BLI sensorgrams show the increase of concentration-dependent target binding aptamers (B) tNSP1, (C) tNSP2, and (D) tNSP3 after being subtracted from the assay buffer with 0 nM N protein as a reference.

the N protein in human saliva and identifying aptamer-protein binding epitopes.

Assessment of tNSP3 aptamer binding to SARS-CoV-2 nucleocapsid in various conditions

With different sources of the wild-type N protein production, the binding affinity of the tNSP3 aptamer to our in-house expressed N protein using *E. coli* was tested under the same conditions, and showed a K_D of 7.52 ± 0.50 nM (Figure S7) compared with the commercial recombinant protein modified with glycosylation and expressed in HEK293 cells. In addition to detecting the N protein obtained from wild-type SARS-CoV-2, an affinity study of our tNSP3 aptamer to the Delta and Omicron variants was also performed. The calculated K_D values of the aptamer to the Delta and Omicron variants were 0.63 ± 0.30 and 3.55 ± 0.20 nM, respectively (Figures 4A and 4B). Taking the safety of the clinical laboratory into consideration, viral inactivation is currently required prior to processing the samples reported by other studies.[37,38] Heat inactivation of SARS-CoV-2 virus has been commonly used to treat the virus-containing serum.[39–41] Thus, the binding affinity of the tNSP3 to the N proteins in the BLI assay buffer (PBST) was investigated after heating the N protein of Delta and Omicron variants at 65°C for 15 min and 80°C for 5 min. The results showed that the incubation of the variant N proteins at 65°C had decreased about 20% and 40% the binding signal of tNSP3 aptamer to the Omicron and Delta variants, respectively. Incubation at 80°C showed a similar effect on the binding affinity to the N protein of the Omicron variant. On the other hand, this resulted in a 60% loss of binding signal to detect the N protein of the Delta variant compared with the binding signals of non-heated N proteins (Figures 4C and 4D).

Detection of SARS-CoV-2 nucleocapsid protein in human saliva using tNSP3 aptamer

To investigate the aptamer-based detection of COVID-19, the tNSP3 aptamer was used to detect the presence of SARS-CoV-2 N protein in human saliva. Concentrated saliva obtained from unspecified-gender pools was diluted in PBST buffer to 10%. The biotinylated aptamer was loaded on a streptavidin biosensor, and the testing saliva sample containing different concentrations of the

Table 1. DNA sequences of truncated N-binding aptamers (tNSP1, tNSP2, and tNSP3) and thermodynamic and kinetic binding parameters of the aptamers to SARS-CoV-2 N protein calculated by using a 1:1 binding model

		Aptamer partitioning in the assay buffer		
Aptamer	Aptamer sequence	K_D (nM)	k_a (1/M·s)	k_d (1/s)
tNSP1 (49 nt)	TAACCACGGCGCAAGCCGGGGTGTACGTGTTATACGTGCGTGTATCGAG	4.53 ± 0.96	4.53×10^{5}	2.05×10^{-3}
tNSP2 (64 nt)	CTGACTGTAACCACGTATTGCGTTCCAGTCCCTATGACCAACGTCACAAT AAGTCGCATAGGTA	3.04 ± 1.53	4.26×10^{5}	1.30×10^{-3}
tNSP3 (44 nt)	CAGCGTCACGTGTTGTTCCCCATTGTACTGATTCGTCGTGGCAT	5.77 ± 1.17	7.20×10^{5}	4.16×10^{-3}
		Aptamer immobilized on the streptavidin biosensors		
Aptamer	Aptamer sequence	K_D (nM)	k_a (1/M·s)	k_d (1/s)
tNSP1 (49 nt)	TAACCACGGCGCAAGCCGGGGTGTACGTGTTATACGTGCGTGTACGAG	4.83 ± 1.09	1.63×10^{5}	7.88×10^{-4}
tNSP2 (64 nt)	CTGACTGTAACCACGTATTGCGTTCCAGTCCCTATGACCAACGTCCAA TAAGTCGCATAGGTA	4.51 ± 1.50	1.13×10^{6}	5.09×10^{-4}
tNSP3 (44 nt)	CAGCGTCACGTGTTGTTCCCCATTGTACTGATTCGTCGTGGCAT	2.91 ± 1.02	1.06×10^{5}	3.87×10^{-4}

N protein was completed with the aptamer. The binding measurement was achieved within 8 min per sample, and the control assay was performed with 10% saliva without N protein. Notably, the tNSP3 aptamer could detect the N protein in the human-pooled saliva with K_D of 50.9 ± 1.26 nM (Figure 5A). The binding of the scrambled DNA to the N protein was not observed. In the controlled experiment, the streptavidin biosensor without tNSP3 aptamer was tested for binding with the highest concentration of N protein (123.04 nM) in the 10% saliva. Accordingly, binding of the N protein to the biosensor was not observed, so the non-specific binding was negligible for this aptamer-based detection. Figure 5B shows the increased binding shifts with concentrations of N protein in the range of 7.79–123.04 nM and the limit of detection (LOD) of 4.5 nM or about 6.77×10^{11} molecules in 1.125 pmol of the N protein.

Identification of nucleocapsid binding epitope-targeting aptamer by nLC-MS/MS

To identify the binding sites of tNSP3 aptamer on the SARS-CoV-2 nucleoprotein, restricted proteolytic digestion of the aptamer-protein complex was performed, and streptavidin magnetic beads were used to pull down peptides bound to the biotinylated aptamer while non-binding peptides were washed out (Figure 6A). The resulting binding peptides were eluted and subjected to nano liquid chromatography-tandem mass spectrometry (nLC-MS/MS) analysis. The intensities of the eluted peptides from the tNSP3 aptamer were compared with a scrambled-DNA control experiment. Abundance differences of individual peptides were compared (Figure 6B) and visualized along the protein sequence (Figure 6C). The eluted peptide at location 376–385 (denoted position in SARS-CoV-2 nucleoprotein, UniProt: P0DTC9) revealed the highest abundance, and this peptide with the amino acid sequence ADETQALPQR (named AA10) was identified as a possible binding epitope. Apart from the AA10 peptide, another peptide appeared at the location of 69–89 with the amino acid sequence GQGVPINTNSSPDDQIGYYRR (named AA21); however, its abundance difference showed a 2-fold lower abundance compared with the AA10 peptide.

Molecular modeling of the aptamer-protein binding interaction

The primary sequence of tNSP3 aptamer was used to predict its secondary structure by the mFold web server[42] (Figure 7A). The corresponding tertiary structure of the tNSP3 was modeled using SimRNA[43] and VMD[44,45] programs. After that, 200-ns molecular dynamics (MD) simulations were performed using GROMACS package[46] to obtain the spatial structure of the tNSP3 aptamer in the solution (Figure 7B). Owing to the high binding affinity of the tNSP3 aptamer, molecular docking for this aptamer was performed to predict the spatial structure of the protein-ligand complex and to reveal the critical nucleotides providing the binding. There is no known experimental structure of the whole N protein to date. In this study, the N protein tertiary structure was predicted by modeling using Iterative Threading ASSEmbly Refinement (I-TASSER) service.[47] The model with the best score referred to a reported modeling template[48] was selected and subjected to 200-ns MD simulations followed by cluster analysis of MD trajectories. The obtained equilibrated structure of the N protein (Figure 6C) was used for molecular docking. Considering AA10 and AA21 peptides as the binding sites on the N protein, molecular docking was performed using HDOCK web server.[49] As a result, the HDOCK program yielded ten different aptamer-protein complexes for both AA10 and AA21 binding sites. The top-scoring binding poses were taken to study aptamer-protein interaction. The models of these binding sites were further refined using MD simulations to obtain the best structures in the solution condition (Figures 6D and 6E). Both considered complexes were stable during 200-ns-long MD simulations and showed no dissociation. Two clusters with a large population in MD simulation were chosen for a more detailed evaluation of residue-nucleotide interaction for each complex. Detailed information on the hydrogen bonds is given in Table S3, and both complexes did not reveal a significant difference in the total number of hydrogen bonds. Different nucleotides are involved in the binding with AA10 and AA21 epitopes. For the AA10-binding epitope, the binding was primarily driven by G15, T17, C18, and C19 nucleotides, whereas the binding of the AA21 epitope was mainly due to T32, A22, and T23 nucleotides. All these nucleotides are located in the loop of the tNSP3 aptamer.

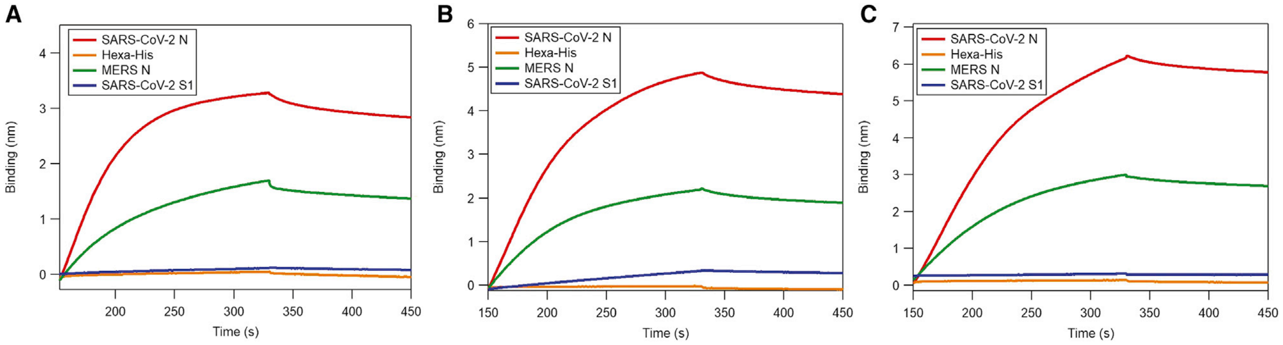

Figure 3. Selectivity of aptamer binding to SARS-CoV-2 N protein

BLI sensorgrams of association and dissociation of the aptamers (A) tNSP1, (B) tNSP2, and (C) tNSP3 with 20 μg/mL SARS-CoV-2 N, 20 μg/mL MERS N, and 20 μg/mL SARS-CoV-S1 proteins in PBST buffer as well as 20 μg/mL hexahistidine peptide as non-specific binding controls.

DISCUSSION

During viral infection, the nucleocapsid (N) protein was reported to possess abundant expression and high immunogenicity in the infected human cells, which is amendable to be targeted at the early stage of viral infection for COVID-19 diagnosis.[50]Therefore, the N protein has been used as a target for aptamer selection to develop an aptamer-based label-free detection method. Several studies have implemented the N protein-binding aptamers to improve the sensitivity of aptamer-based detections.[51,52]This work's novelty is selecting DNA aptamers binding to SARS-CoV-2 nucleocapsid via asymmetric emulsion PCR, identifying the aptamer-N protein binding epitopes, and using the aptamers in BLI-based detection of the N protein in human saliva. We discovered the novel sequences of DNA aptamers from a synthetic DNA library using the N protein of wild-type SARS-CoV-2 as a binding target. The selection was stopped at the sixth round because of emerging PCR by-products caused by PCR artifacts, such as a primer-dimer and primer partially hybridized DNA, which might interfere with the binding efficiency between the targeted DNA aptamers and the N protein.[53,54] The most enriched sequences over all rounds were considered to retrieve aptamers with high affinity and specificity.

The truncated structures of these selected aptamers (tNSP1, tNSP2, and tNSP3) evinced a high binding affinity to the N protein with a similar range of K_D values. The binding affinity of truncated structures was not significantly affected by trimming out the primer-binding region. While aptamers with their full length may form various unfavorable secondary structures, the smaller size of aptamers renders the higher binding affinity due to less steric hindrance of the aptamer structures.[55,56] These findings were consistent with previously reported studies.[57,58] The three truncated aptamers did not exhibit cross-reactivity to the SARS-CoV-2 S1 subunit and hexahistidine peptide. The aptamers revealed 2-fold decreased binding to the nucleocapsid protein of MERS coronavirus. This can be explained by the high level of similarity of peptide sequences between nucleocapsid of SARS-CoV-2 and MERS, as previously reported.[59,60] To prevent false-positive detection of our aptamer-based BLI for MERS N protein, a cutoff diagnostic threshold may be set up by using the binding signal of the aptamer to the MERS N protein as a background signal. With tNSP3 possessing a K_D value below 4 nM to wild type as well as Omicron and a K_D value of 0.6 nM to the Delta variant, the epitope of N protein binding to the tNSP3 was identified. Notably, the aptamer-binding epitope was localized in the C-terminal domain with high binding affinity, which can explain the specific interaction of the tNSP3 aptamer with SARS-CoV-2 over MERS coronaviruses due to the dissimilarity of the amino acid sequences of the N protein. In addition, the interaction of the AA10-binding site with the tNSP3 was modeled by MD simulation. Interestingly, there are no identical nucleotide-amino acid pairs in the aptamer-protein complexes, assuming that an individual nucleotide may preferably bind to the specific amino acids on the binding sites. Even though the tNSP3 aptamer showed partial binding to the AA21 peptide localized in the RNA-binding domain, this might be a non-specific interaction mainly through electrostatic interactions between positive residues on the N protein and a phosphate backbone of the DNA aptamer. The limitation of the peptide-binding method lies in potentially different folding and conformational dynamics of the full-sized protein and digested peptides. This difference can change binding kinetics and decrease the affinity of aptamers.

In addition, the performance of the tNSP3 aptamer was assessed by detecting the SARS-CoV-2 N protein spiked in human saliva. Owing to the high complexity of saliva samples, including metabolites and proteins, it affected the aptamer-protein interaction and increased K_D up to 50 nM. The tNSP3 aptamer implemented on BLI has an LOD in the nanomolar range comparable with other aptamer-based detections previously reported in the literature.[61–63] Nevertheless, the detection of SARS-CoV-2 N protein using an aptamer-based biosensor still requires sample processing, which has been commonly used in the standard clinical procedure.[64] Once the viral particles are lysed and inactivated with heat treatment, the N protein is released from the viral RNA, which is necessary for aptasensors to detect this protein.

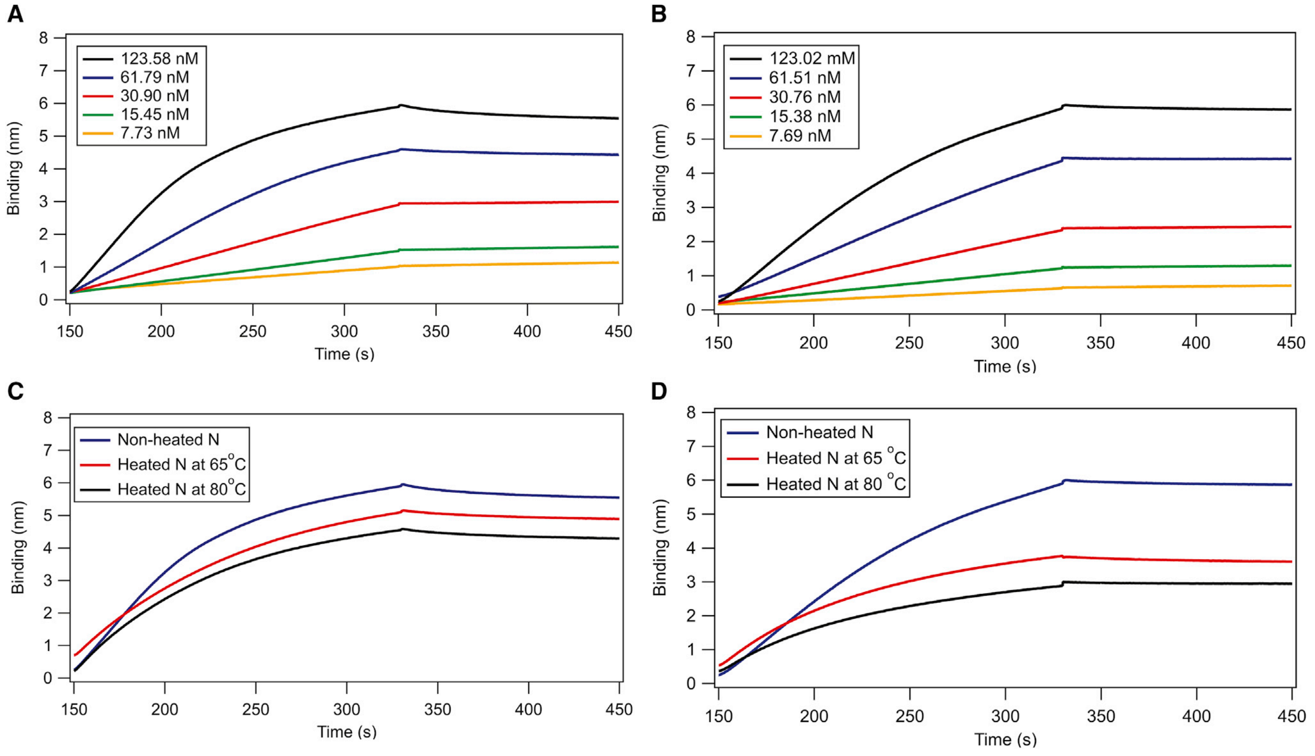

Figure 4. Detection of SARS-CoV-2 N protein of Delta and Omicron variants by tNSP3-based BLI

BLI sensorgrams showing the increased signals of concentration-dependent target N proteins of (A) Omicron and (B) Delta variant binding to the tNSP3 aptamer after being subtracted from the assay buffer with 0 nM N protein as a reference. Under heat treatment the binding affinity of tNSP3 aptamer to the N proteins of (C) Omicron and (D) Delta variants incubated at 65°C for 15 min and 80°C for 5 min.

In conclusion, we discovered novel DNA aptamers binding to the SARS-CoV-2 nucleocapsid protein and its variants, Delta and Omicron. The selected aptamer named tNSP3 implemented onto the BLI biosensor platform successfully detected the N protein in human saliva. With the high specificity of the tNSP3 aptamer to the N protein, the cross-reactivity to SARS-CoV-2 S1 was negligible; however, the binding of this aptamer to MERS N protein was observed with a 50% reduction. Future work will aim to apply our aptamer-based approach to clinical samples.

MATERIALS AND METHODS

Asymmetric-emulsion PCR-SELEX

The selection of the N protein-binding aptamers was initiated by heating 2 nmol of DNA library: 5′-CTC CTC TGA CTG TAA CCA CG-(N40)-GCA TAG GTA GTC CAG AAG CC-3′ (Integrated DNA Technologies, Coralville, IA) in the selection buffer (Dulbecco's PBS [DPBS], with 0.5 mM $MgCl_2$, 1 mM $CaCl_2$, 2 μg/μL BSA, and 0.2 μg/μL tRNA) at 95°C for 5 min, then cooled on ice for 10 min. First, a counter-selection was performed by directly adding the heated DNA library mixture to an Ni-NTA HisSorp strip (Qiagen, Germantown, MD) without adding SARS-CoV-2 N protein followed by incubation for 30 min at room temperature in the first round of selection. Additionally, the counter-selections were performed in the fourth and sixth rounds, in which the enriched DNA pools from the previous rounds of selection were incubated with SARS-CoV-2 S1 (ACRObiosystems, Newark, DE) coated on the Ni-NTA strips. After the counter-selection was completed, the positive selection was performed by transferring the unbound DNA mixture from the counter-selection to an Ni-NTA HisSorb strip coated with 20 μg/mL his-tagged N protein (ACRObiosystems) and incubating for 1 h at room temperature. The unbound DNAs were then washed twice with 500 μL of the washing buffer (DPBS with 0.5 mM $MgCl_2$, 1 mM $CaCl_2$, and 0.05% Tween 20). The N-protein-binding DNAs on the strip were eluted by incubating with nuclease-free water at 95°C for 10 min. The stringency of the six-round selection was increased by an additional number of washings and the decrease of incubation time of the DNA-protein mixture for 30 min in the fourth to sixth rounds. Also, the ratio of protein to DNA was decreased by two times for each round. Thereafter, the eluted DNA was mixed with an asymmetric PCR mixture (forward-to-reverse primer ratio of 20:1), and the PCR mixture was added dropwise to the emulsion oil while stirring. The optimized PCR amplification was performed for 25 cycles (98°C for 30 s; 25 cycles at 98°C for 30 s, 56°C for 15 s, 72°C for 15 s; and hold at 4°C), and the amplicons were separated from the oil phase by adding 1 mL of isopropanol and centrifuging at 15,000 × *g* for 5 min. The aqueous phase at the bottom layer,

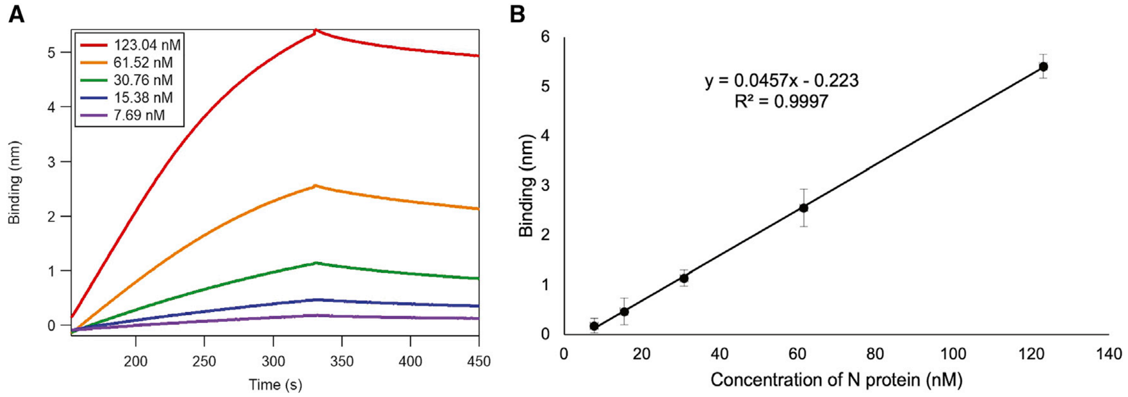

Figure 5. Aptamer-based BLI detection of SARS-CoV-2 N protein in human saliva

(A) BLI sensorgram displaying the binding assay of tNSP3 with the N protein spiked in human saliva with 2-fold dilution to determine the K_D. (B) Linear regression curve of the binding shifts corresponding to the N protein in the concentration range of 7.69–123.04 nM.

containing the amplified ssDNA, was then purified with a PCR cleanup kit (New England BioLabs, Ipswich, MA).

Next-generation sequencing and data analysis

After six selection rounds, each ssDNA pool was reamplified by symmetric PCR and purified before NGS. The fastq raw data were analyzed following the guideline from FASTAptamer,[36] as shown in Figure S2. The data representing each aptamer pool were categorized into its cluster, followed by using the FASTAptamer-enrich tool to retrieve the most enriched sequences. After the aptamer sequences were identified, the multiple sequence alignment tool on Clustal Omega was used to evaluate the similarity and differentiation of individual aptamer sequences. The secondary structures of the selected aptamers were then predicted using the RNAstructure web server.[38]

Evaluation of nucleocapsid aptamer binding affinity by biolayer interferometry

The binding affinity of aptamer pools and SARS-CoV-2 nucleocapsid protein (ACRObiosystems) was performed using Ni-NTA biosensors and a BLI instrument named Octet N1 (Sartorius, Bohemia, NY). First, the DNA-enriched pools were prepared in the assay buffer (DPBS with 0.5 mM $MgCl_2$ and 1 mM $CaCl_2$, pH 7.4) at a concentration of 500 nM and heated at 95°C for 5 min, then ice-cooled. The binding assay was performed in 0.5-mL light-blocking tubes at 25°C with a total volume of 250 μL. In the next steps, the his-tagged N protein was loaded on Ni-NTA biosensor tips. During the binding assay, a baseline was established in the binding buffer followed by monitoring of the association between the DNA-enriched pools and the N protein on the biosensors. The association step was performed with six different concentrations (0, 12.5, 25, 50,100, and 200 nM) of NSP1, NSP2, and NSP3 aptamers. The assay was carried on Octet N1 in the DPBS buffer at 25°C with a 30-s baseline, 100 s for association, and 150 s for dissociation, and glycine-HCl (pH 1.7) was used for biosensor regeneration. For the non-specific binding test, the 80-nt-scrambled DNA (Sc80) was used as a negative control. The published aptamers A48 and A58 were included in this experiment as positive controls.

To imitate the COVID-19 diagnosis, the binding assay was performed by immobilizing the aptamers on the biosensor tips to detect the presence of the N protein in the assay buffer. The serial dilutions of the N protein (7.69, 15.38, 30.76, 61.52, and 123.04 nM) were spiked in the optimized assay buffer (PBST, DPBS with 0.5 mM $MgCl_2$ and 1 mM $CaCl_2$ [pH 7.4], 0.2% BSA and 0.05% Tween 20 added). The detection was performed by dipping a streptavidin biosensor (Sartorius, Bohemia, NY), which was immobilized with the biotinylated aptamer, into the N protein spiked PBST buffer. The assay was carried out at 25°C with 180 s for association and 120 s for dissociation. The assay buffer was used to wash out the unbound biomolecules from the biosensor tip during the baseline step for 60 s. The K_D of each aptamer was calculated by Octet N1 software (version 1.3.0.5) using a 1:1 global binding model. Additionally, the binding affinity of tNSP3 aptamer to our in-house expressed SARS-CoV-2 nucleocapsid protein and the N proteins of Delta (7.69, 15.38, 30.76, 61.51, and 123.02 nM) as well as Omicron (7.73, 15.45, 30.90, 61.79, and 123.58 nM) variants was also performed under the same conditions described above.

To study the effect of heat inactivation on the aptamer-based detection of N proteins, the biotinylated tNSP3 aptamer was immobilized on the streptavidin biosensor tips, and the binding assay of the aptamer to the N proteins of Delta (123.02 nM) and Omicron (123.58 nM) variants that were heated at two different temperature conditions (65°C for 15 min and 80°C for 5 min) was performed on Octet N1 with the same assay buffer mentioned above.

Cross-reactivity of aptamer binding to N protein

The selectivity of three aptamers (tNSP1, tNSP2, and tNSP3) to the N protein was examined by investigating the binding affinity of the aptamers to other viral proteins, including SARS-CoV-2 S1 protein and

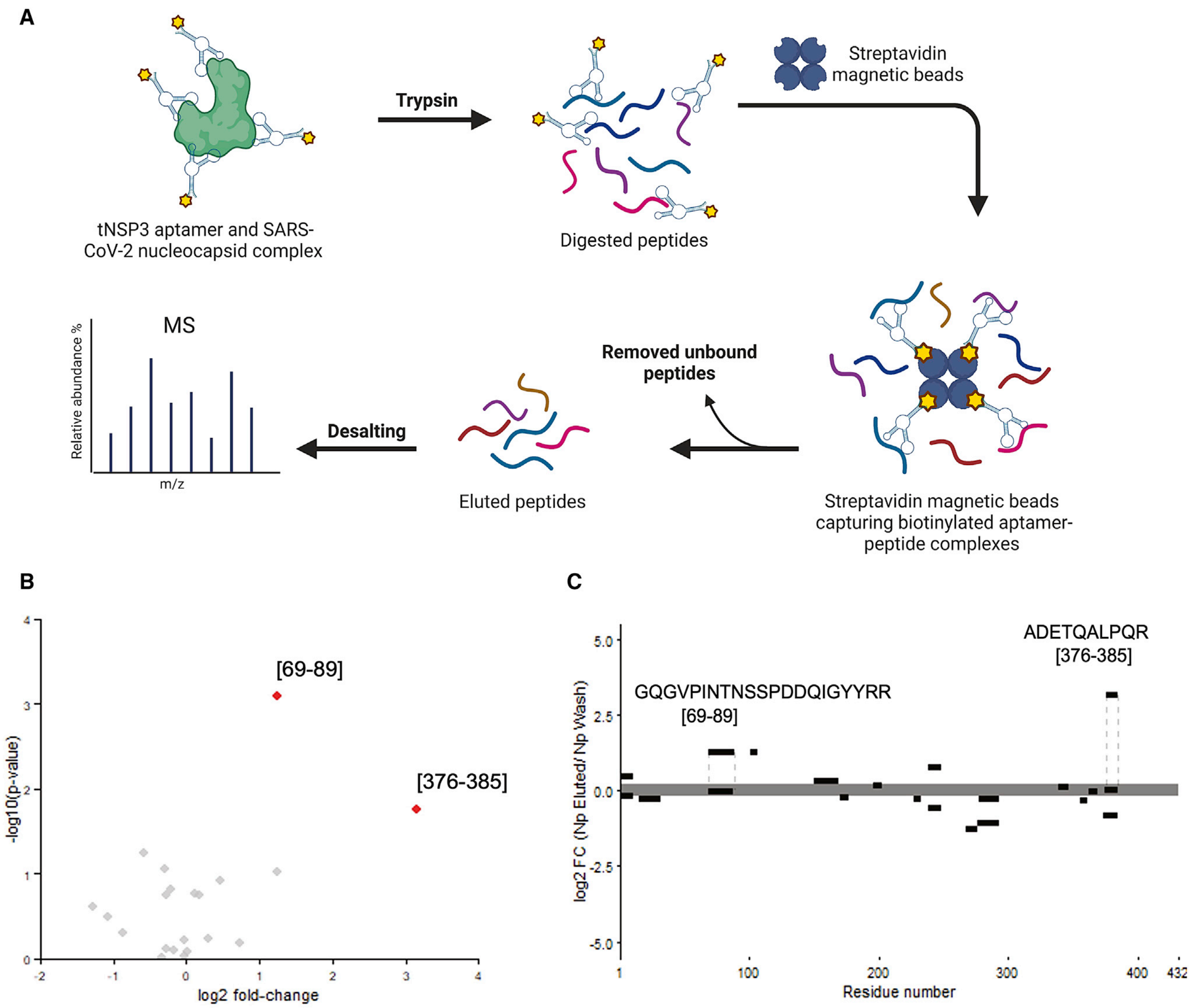

Figure 6. Identification of epitope peptide binding to tNSP3 aptamer by nLC-MS/MS

(A) Experimental scheme of pull-down assay used for identifying aptamer-binding peptides. (B) Volcano plot showing fold change (x axis) and statistical significance (y axis) of individual peptides based on label-free quantification intensity. Red points indicate peptides of interest appearing in the eluted peptides from tNSP3 aptamer in significantly higher abundance. (C) Peptide plots of log_2 fold-change differences of peptides visualized along the SARS-CoV-2 N sequence. The peptides significantly more abundant in the fractions eluted from tNSP3 aptamer were identified by a paired t test and annotated by dotted lines, $p < 0.05$.

the nucleocapsid protein of MERS coronavirus (ACRObiosystems). Following the optimized condition of the binding assay as previously mentioned, cross-reactivity was performed in the assay buffer (PBST, DPBS with 0.5 mM $MgCl_2$ and 1 mM $CaCl_2$ [pH 7.4], 0.2% BSA and 0.05% Tween 20 added) on Octet N1 at 25°C with a 60-s baseline, 180 s for association, and 120 s for dissociation. Each biotinylated aptamer (400 nM) was immobilized on the streptavidin biosensor tips. The binding affinity of the aptamers to the non-targeted proteins (SARS-CoV-2 S1 and MERS N proteins) was compared with the SARS-CoV-2 N protein at varying concentrations (7.69, 15.38, 30.76, 61.52, and 123.04 nM), and the apparent K_D values were calculated using a 1:1 global binding model as mentioned above. To verify non-specific binding to the protein tag, a hexahistidine peptide was also included as a control in the binding assay.

Detection of SARS-CoV-2 nucleocapsid protein in human saliva using tNSP3 aptamer

The serial dilutions of N protein (7.69, 15.38, 30.76, 61.52, and 123.04 nM) were spiked in 10% saliva assay buffer, which was prepared by diluting the concentrated human saliva (BioIVT, Westbury, NY) in PBST buffer (DPBS with 0.5 mM $MgCl_2$, 1 mM $CaCl_2$, 0.2% BSA, and 0.05% Tween 20 [pH 7.4]). The detection was performed

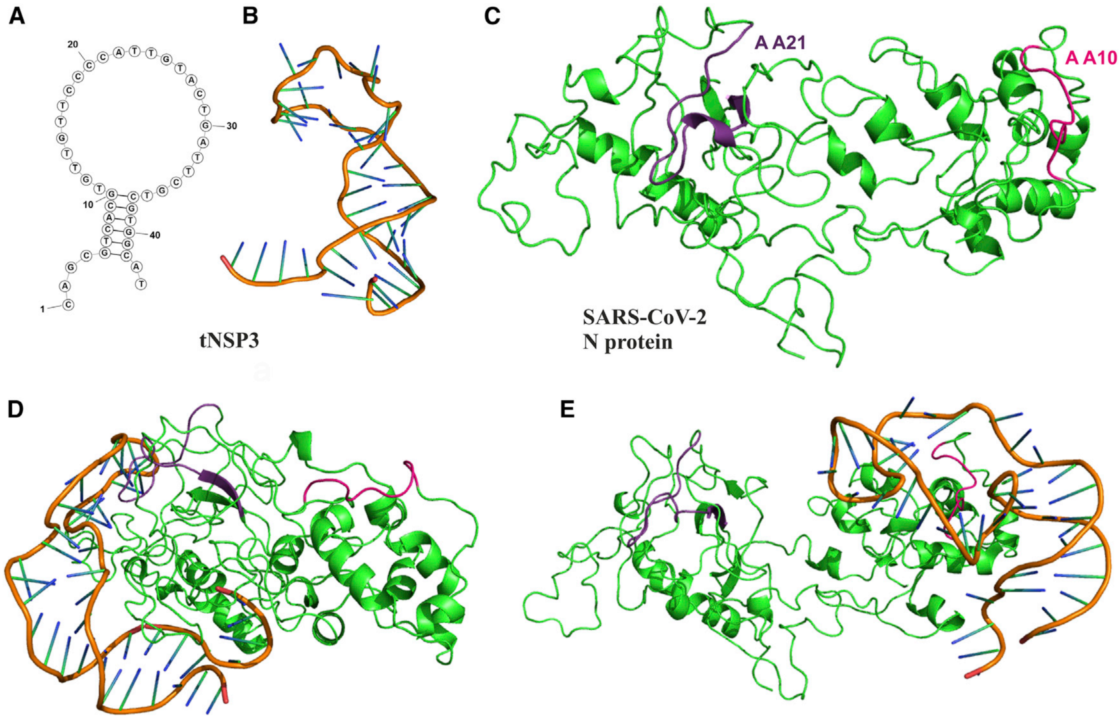

Figure 7. Structure of tNSP3-N protein complexes

(A) Secondary and (B) tertiary structures of tNSP3 aptamer. (C) Tertiary structure of SARS-CoV-2 nucleocapsid protein. (D and E) Complexes of tNSP3 binding to AA21 and AA10 motifs on the N protein. The AA21 and AA10 peptide motifs are colored purple and red, respectively.

by immobilizing the biotinylated tNSP3 aptamer on a streptavidin biosensor tip. As mentioned in the previous assay, the N protein spiked in 10% saliva was detected on Octet N1, and the 10% saliva assay buffer without adding the N protein was used as the background signal. The apparent K_D of the aptamer was calculated with Octet N1 software (version 1.3.0.5) using a 1:1 global binding model, and the LOD was also determined based on the standard deviation (SD) of the binding curves and the slope of binding (nm) versus concentration of the N protein (nM): LOD = 3.3SD/slope.[65]

Identification of SARS-CoV-2 nucleocapsid and aptamer-binding epitopes using nLC-MS/MS

First, 20 pmol of SARS-CoV-2 nucleocapsid in 25 mM HEPES (pH 7.5) was incubated with 100 pmol biotinylated tNSP3 aptamer heat-folded in the incubation buffer (DPBS with 0.5 mM $MgCl_2$, 1 mM $CaCl_2$, and 0.2 μg/μL tRNA) for 30 min at 25°C. The DNA-protein mixture was then transferred to a Microcon-30kDa filter (Millipore, Burlington, MA) and centrifuged at 14,000 $\times$ g for 10 min to clean up the unbound DNAs. The solution of aptamer-protein complexes retained on the filter was transferred to a 1.5-mL microcentrifuge tube. The aptamer-bound protein complexes were digested by adding 1.5 μL of 0.2 μg/μL trypsin/Lys-C followed by incubation at 37°C for 30 min. The digested aptamer-protein complexes were added to the pre-washed Dynabeads MyOne streptavidin magnetic beads (Invitrogen, Waltham, MA) according to the manufacturer's protocol. In brief, the biotinylated tNSP3 aptamer-digested peptide complexes were incubated with the streptavidin magnetic beads on a rotator at room temperature for 15 min, and the unbound biotinylated aptamer was washed out twice with the washing buffer (DPBS with 0.5 mM $MgCl_2$ and 1 mM $CaCl_2$). Next, 100 μL of 8 M urea was added and incubated for 10 min in order to elute the aptamer-bound peptides at room temperature. The eluted peptides were collected, and 2 μL of formic acid was added to the sample which was stored at −20°C until further processing. The digested peptides were cleaned up by desalting on C18 stage tips (TopTip) before injection into the nLC-MS/MS system. Another control sample was initially performed by incubating the heat-folded scrambled DNA (Sc80) with the N protein for 30 min at 25°C before transferring the aptamer-protein complexes to the Microcon-30kDa filter. The digestion of aptamer-protein complexes was performed using trypsin as described previously. Each sample was prepared in triplicates for statistical analysis.

Epitope identification by proteomics data analysis

MS raw files were analyzed with MaxQuant (version 2.0.3.0)[66] and the Andromeda search engine.[67] Peptides were searched against a human UniProt FASTA file containing 20,408 entries (April 21, 2021) and a default contaminants database. Default parameters were used if not mentioned otherwise. N-terminal acetylation and methionine oxidation were set as variable modifications, and

cysteine carbamidomethylation was set as a fixed modification. A minimum peptide length of 6 amino acids was required. The false discovery rate was set to 0.01 for the protein and peptide levels, determined by searching against a reverse sequence database. Enzyme specificity was set as C-terminal to arginine and lysine with a maximum of two missed cleavages. Peptides were identified with an initial precursor mass deviation of up to 10 ppm and a fragment mass deviation of 0.5 Da. The "match between runs" algorithm in MaxQuant[68] was performed between all samples to increase the peptide identification rate. Proteins and peptides matching the reverse database were discarded. For label-free protein quantitation (LFQ), a minimum ratio count of 2 was required.[69] To identify the aptamer epitope on the N protein, the $\log_2$ fold change and a p value for each peptide were computed from the LFQ intensity between eluted fractions obtained from the tNSP3 aptamer and the scrambled DNA. Peptides significantly more abundant ($p < 0.05$) in the fraction eluted from the aptamer were considered potential epitope peptides. The sequence position of individual peptides was visualized with R and the pOmics and ggplot2 packages.

Molecular dynamics simulation of peptide-aptamer interaction

The secondary structures of the aptamers were predicted on the basis of their sequences using the MFold web server.[70] Protein folding was performed using the I-TASSER server.[47] A model with the highest C score was chosen for further refinement by MD simulations to improve structure quality. Aptamer-protein complexes were obtained by the HDOCK molecular docking web server.[49] MD simulations of the aptamers, N protein, and aptamer-protein complexes were conducted in the same way as previously described[71] by using GROMACS 2019.8 software[46] with the Amber14sb force field[72] and the TIP3P model[73] for water. The negative charge of the complexes was neutralized with Na^+ ions. Na^+ and Cl^- ions were then added to the system at the concentration of 0.15 M. After MD simulations, clusters of structures and their centers were computed using the quality threshold algorithm[74] with the VMD program.[44]

DATA AVAILABILITY

The authors confirm that the data supporting the findings of this study are available within the article and its supplemental information. Additional raw data that support the findings of this study can be made available from the corresponding author upon reasonable request.

SUPPLEMENTAL INFORMATION

Supplemental information can be found online at https://doi.org/10.1016/j.omtn.2023.02.010.

ACKNOWLEDGMENTS

M.V.B. thanks the Canadian Institutes of Health Research grant OV1-170353 for providing financial support. Molecular modeling and docking were supported by a grant from the Russian Science Foundation (project number 21-73-20240) for A.S.K. S.P. is thankful to Dr. Bob Dass, Dylan Tanner, and Dr. Degang Liu, Sartorius for generously providing excellent technical training and consumable support for binding assay on BLI, and Aldo Jordan for assisting with creating the figures. The authors also thank John L. Holmes's mass spectrometry facility for providing access to perform nLC-MS/MS. Lastly, the authors thank the JCSS Joint Super Computer Center of the Russian Academy of Sciences for providing supercomputers for computer simulations.

AUTHOR CONTRIBUTIONS

S.P. designed the experiments, selected aptamers, analyzed NGS data, performed the binding affinity assays, and collected and analyzed the data. N.H., P.V.A., I.A.S., F.N.T., and A.S.K. designed the software and analyzed the data. E.Z. expressed SARS-CoV-2 nucleocapsid. Z.M. analyzed the digested peptides on nLC-MS/MS and collected the data. S.P., N.H., E.Z., and P.V.A. wrote the manuscript. S.P., E.Z., N.H., Z.M., and M.V.B contributed to reviewing and editing the manuscript. M.V.B. supervised and processed fund acquisition. All authors reviewed the manuscript.

DECLARATION OF INTERESTS

There authors declare no competing interests.

REFERENCES

1. Li, G., and De Clercq, E. (2020). Therapeutic options for the 2019 novel coronavirus (2019-nCoV). Nat. Rev. Drug Discov. *19*, 149–150.
2. World Health Organization (2022). WHO Coronavirus (COVID-19) Dashboard. https://covid19.who.int/.
3. Udugama, B., Kadhiresan, P., Kozlowski, H.N., Malekjahani, A., Osborne, M., Li, V.Y.C., Chen, H., Mubareka, S., Gubbay, J.B., and Chan, W.C.W. (2020). Diagnosing COVID-19: the disease and tools for detection. ACS Nano *14*, 3822–3835.
4. Yüce, M., Filiztekin, E., and Özkaya, K.G. (2021). COVID-19 diagnosis —a review of current methods. Biosens. Bioelectron. *172*, 112752.
5. Lu, R., Zhao, X., Li, J., Niu, P., Yang, B., Wu, H., Wang, W., Song, H., Huang, B., Zhu, N., et al. (2020). Genomic characterisation and epidemiology of 2019 novel coronavirus: implications for virus origins and receptor binding. Lancet *395*, 565–574.
6. Yang, H., and Rao, Z. (2021). Structural biology of SARS-CoV-2 and implications for therapeutic development. Nat. Rev. Microbiol. *19*, 685–700.
7. Smyrlaki, I., Ekman, M., Lentini, A., Rufino de Sousa, N., Papanicolaou, N., Vondracek, M., Aarum, J., Safari, H., Muradrasoli, S., Rothfuchs, A.G., et al. (2020). Massive and rapid COVID-19 testing is feasible by extraction-free SARS-CoV-2 RT-PCR. Nat. Commun. *11*, 4812.
8. Dong, L., Zhou, J., Niu, C., Wang, Q., Pan, Y., Sheng, S., Wang, X., Zhang, Y., Yang, J., Liu, M., et al. (2021). Highly accurate and sensitive diagnostic detection of SARS-CoV-2 by digital PCR. Talanta *224*, 121726.
9. Vogels, C.B.F., Brito, A.F., Wyllie, A.L., Fauver, J.R., Ott, I.M., Kalinich, C.C., Petrone, M.E., Casanovas-Massana, A., Catherine Muenker, M., Moore, A.J., et al. (2020). Analytical sensitivity and efficiency comparisons of SARS-CoV-2 RT–qPCR primer–probe sets. Nat. Microbiol. *5*, 1299–1305.
10. Hwang, Y.-C., Lu, R.-M., Su, S.-C., Chiang, P.-Y., Ko, S.-H., Ke, F.-Y., Liang, K.-H., Hsieh, T.-Y., and Wu, H.-C. (2022). Monoclonal antibodies for COVID-19 therapy and SARS-CoV-2 detection. J. Biomed. Sci. *29*, 1.
11. Yakoh, A., Pimpitak, U., Rengpipat, S., Hirankarn, N., Chailapakul, O., and Chaiyo, S. (2021). Paper-based electrochemical biosensor for diagnosing COVID-19: detection of SARS-CoV-2 antibodies and antigen. Biosens. Bioelectron. *176*, 112912.
12. U.S. Food and Drug Administration (2022). In Vitro Diagnostics EUAs - Antigen Diagnostic Tests for SARS-CoV-2. https://www.fda.gov/medical-devices/coronavirus-disease-2019-covid-19-emergency-use-authorizations-medical-devices/in-vitro-diagnostics-euas-antigen-diagnostic-tests-sars-cov-2.

13. Machado, B.A.S., Hodel, K.V.S., Barbosa-Júnior, V.G., Soares, M.B.P., and Badaró, R. (2020). The main molecular and serological methods for diagnosing COVID-19: an overview based on the literature. Viruses *13*, 40.

14. Wang, D., He, S., Wang, X., Yan, Y., Liu, J., Wu, S., Liu, S., Lei, Y., Chen, M., Li, L., et al. (2020). Rapid lateral flow immunoassay for the fluorescence detection of SARS-CoV-2 RNA. Nat. Biomed. Eng. *4*, 1150–1158.

15. Wang, Q., Tan, L., Wu, W., Tang, S., Xiong, Z., and Zheng, S. (2020). Evaluation of Nucleocapsid and Spike Protein-Based Enzyme-Linked Immunosorbent Assays for Detecting Antibodies against SARS-CoV-2. J Clin Microbiol. *58*. e00461–20.

16. Chen, Z., Zhang, Z., Zhai, X., Li, Y., Lin, L., Zhao, H., Bian, L., Li, P., Yu, L., Wu, Y., and Lin, G. (2020). Rapid and sensitive detection of anti-SARS-CoV-2 IgG, using lanthanide-doped nanoparticles-based lateral flow immunoassay. Anal. Chem. *92*, 7226–7231.

17. Liu, G., and Rusling, J.F. (2021). COVID-19 antibody tests and their limitations. ACS Sens. *6*, 593–612.

18. Poolsup, S., and Kim, C.-Y. (2017). Therapeutic applications of synthetic nucleic acid aptamers. Curr. Opin. Biotechnol. *48*, 180–186.

19. Li, L., Xu, S., Yan, H., Li, X., Yazd, H.S., Li, X., Huang, T., Cui, C., Jiang, J., and Tan, W. (2021). Nucleic acid aptamers for molecular diagnostics and therapeutics: advances and perspectives. Angew. Chem., Int. Ed. Engl. *60*, 2221–2231.

20. Tuerk, C., and Gold, L. (1990). Systematic evolution of ligands by exponential enrichment: RNA ligands to bacteriophage T4 DNA polymerase. Science *249*, 505–510.

21. Ellington, A.D., and Szostak, J.W. (1990). In vitro selection of RNA molecules that bind specific ligands. Nature *346*, 818–822.

22. Cho, S.-J., Woo, H.-M., Kim, K.-S., Oh, J.-W., and Jeong, Y.-J. (2011). Novel system for detecting SARS coronavirus nucleocapsid protein using an ssDNA aptamer. J. Biosci. Bioeng. *112*, 535–540.

23. Zou, X., Wu, J., Gu, J., Shen, L., and Mao, L. (2019). Application of aptamers in virus detection and antiviral therapy. Front. Microbiol. *10*, 1462.

24. Song, Y., Song, J., Wei, X., Huang, M., Sun, M., Zhu, L., Lin, B., Shen, H., Zhu, Z., and Yang, C. (2020). Discovery of aptamers targeting the receptor-binding domain of the SARS-CoV-2 spike glycoprotein. Anal. Chem. *92*, 9895–9900.

25. Schmitz, A., Weber, A., Bayin, M., Breuers, S., Fieberg, V., Famulok, M., and Mayer, G. (2021). A SARS-CoV-2 spike binding DNA aptamer that inhibits pseudovirus infection by an RBD-independent mechanism. Angew. Chem., Int. Ed. Engl. *60*, 10279–10285.

26. Liu, X., Wang, Y.L., Wu, J., Qi, J., Zeng, Z., Wan, Q., Chen, Z., Manandhar, P., Cavener, V.S., Boyle, N.R., et al. (2021). Neutralizing aptamers block S/RBD-ACE2 interactions and prevent host cell infection. Angew. Chem., Int. Ed. Engl. *60*, 10273–10278.

27. Savastano, A., Ibáñez de Opakua, A., Rankovic, M., and Zweckstetter, M. (2020). Nucleocapsid protein of SARS-CoV-2 phase separates into RNA-rich polymerase-containing condensates. Nat. Commun. *11*, 6041.

28. Chen, Z., Wu, Q., Chen, J., Ni, X., and Dai, J. (2020). A DNA aptamer based method for detection of SARS-CoV-2 nucleocapsid protein. Virol. Sin. *35*, 351–354.

29. Che, X.-Y., Hao, W., Wang, Y., Di, B., Yin, K., Xu, Y.-C., Feng, C.-S., Wan, Z.-Y., Cheng, V.C.C., and Yuen, K.-Y. (2004). Nucleocapsid protein as early diagnostic marker for SARS. Emerg. Infect. Dis. *10*, 1947–1949.

30. Zhang, L., Fang, X., Liu, X., Ou, H., Zhang, H., Wang, J., Li, Q., Cheng, H., Zhang, W., and Luo, Z. (2020). Discovery of sandwich type COVID-19 nucleocapsid protein DNA aptamers. Chem. Commun. *56*, 10235–10238.

31. Ge, C., Feng, J., Zhang, J., Hu, K., Wang, D., Zha, L., Hu, X., and Li, R. (2022). Aptamer/antibody sandwich method for digital detection of SARS-CoV2 nucleocapsid protein. Talanta *236*, 122847.

32. Ramanathan, S., Gopinath, S.C.B., Ismail, Z.H., Md Arshad, M.K., and Poopalan, P. (2022). Aptasensing nucleocapsid protein on nanodiamond assembled gold interdigitated electrodes for impedimetric SARS-CoV-2 infectious disease assessment. Biosens. Bioelectron. *197*, 113735.

33. Chen, R., Kan, L., Duan, F., He, L., Wang, M., Cui, J., Zhang, Z., and Zhang, Z. (2021). Surface plasmon resonance aptasensor based on niobium carbide MXene quantum dots for nucleocapsid of SARS-CoV-2 detection. Mikrochim. Acta *188*, 316.

34. Berezovski, M.V., Lechmann, M., Musheev, M.U., Mak, T.W., and Krylov, S.N. (2008). Aptamer-facilitated biomarker discovery (AptaBiD). J. Am. Chem. Soc. *130*, 9137–9143.

35. Müller-Esparza, H., Osorio-Valeriano, M., Steube, N., Thanbichler, M., and Randau, L. (2020). Bio-layer interferometry analysis of the target binding activity of CRISPR-Cas effector complexes. Front. Mol. Biosci. *7*, 98.

36. Alam, K.K., Chang, J.L., and Burke, D.H. (2015). FASTAptamer: a bioinformatic toolkit for high-throughput sequence analysis of combinatorial selections. Mol. Ther. Nucleic Acids *4*, e230.

37. Madeira, F., Pearce, M., Tivey, A.R.N., Basutkar, P., Lee, J., Edbali, O., Madhusoodanan, N., Kolesnikov, A., and Lopez, R. (2022). Search and sequence analysis tools services from EMBL-EBI in 2022. Nucleic Acids Res. *50*, W276–W279.

38. Reuter, J.S., and Mathews, D.H. (2010). RNAstructure: software for RNA secondary structure prediction and analysis. BMC Bioinf. *11*, 129.

39. Batéjat, C., Grassin, Q., Manuguerra, J.C., and Leclercq, I. (2021). Heat inactivation of the severe acute respiratory syndrome coronavirus 2. J. Biosaf. Biosecur. *3*, 1–3.

40. Patterson, E.I., Prince, T., Anderson, E.R., Casas-Sanchez, A., Smith, S.L., Cansado-Utrilla, C., Solomon, T., Griffiths, M.J., Acosta-Serrano, Á., Turtle, L., et al. (2020). Methods of inactivation of SARS-CoV-2 for Downstream biological assays. J. Infect. Dis. *222*, 1462–1467.

41. Delpuech, O., Douthwaite, J.A., Hill, T., Niranjan, D., Malintan, N.T., Duvoisin, H., Elliott, J., Goodfellow, I., Hosmillo, M., Orton, A.L., et al. (2022). Heat inactivation of clinical COVID-19 samples on an industrial scale for low risk and efficient high-throughput qRT-PCR diagnostic testing. Sci. Rep. *12*, 2883.

42. Owczarzy, R., Tataurov, A.V., Wu, Y., Manthey, J.A., McQuisten, K.A., Almabrazi, H.G., Pedersen, K.F., Lin, Y., Garretson, J., McEntaggart, N.O., et al. (2008). IDT SciTools: a suite for analysis and design of nucleic acid oligomers. Nucleic Acids Res. *36*, W163–W169.

43. Boniecki, M.J., Lach, G., Dawson, W.K., Tomala, K., Lukasz, P., Soltysinski, T., Rother, K.M., and Bujnicki, J.M. (2016). SimRNA: a coarse-grained method for RNA folding simulations and 3D structure prediction. Nucleic Acids Res. *44*, e63.

44. Humphrey, W., Dalke, A., and Schulten, K. (1996). VMD: visual molecular dynamics. J. Mol. Graph. *14*, 33–38. 27-28.

45. Jeddi, I., and Saiz, L. (2017). Three-dimensional modeling of single stranded DNA hairpins for aptamer-based biosensors. Sci. Rep. *7*, 1178.

46. Abraham, M.J., Murtola, T., Schulz, R., Páll, S., Smith, J.C., Hess, B., and Lindahl, E. (2015). GROMACS: high performance molecular simulations through multi-level parallelism from laptops to supercomputers. SoftwareX *1–2*, 19–25.

47. Yang, J., Yan, R., Roy, A., Xu, D., Poisson, J., and Zhang, Y. (2015). The I-TASSER Suite: protein structure and function prediction. Nat. Methods *12*, 7–8.

48. Sankararaman, S., Hamre, J., 3rd, Almsned, F., Aljouie, A., Bokhari, Y., Alawwad, M., Alomair, L., and Jafri, M.S. (2022). Active site prediction of phosphorylated SARS-CoV-2 N-Protein using molecular simulation. Inform. Med. Unlocked *29*, 100889.

49. Yan, Y., Tao, H., He, J., and Huang, S.-Y. (2020). The HDOCK server for integrated protein-protein docking. Nat. Protoc. *15*, 1829–1852.

50. Diao, B., Wen, K., Zhang, J., Chen, J., Han, C., Chen, Y., Wang, S., Deng, G., Zhou, H., and Wu, Y. (2021). Accuracy of a nucleocapsid protein antigen rapid test in the diagnosis of SARS-CoV-2 infection. Clin. Microbiol. Infect. *27*, 289.e1–289.e4.

51. Singh, N.K., Ray, P., Carlin, A.F., Magallanes, C., Morgan, S.C., Laurent, L.C., Aronoff-Spencer, E.S., and Hall, D.A. (2021). Hitting the diagnostic sweet spot: point-of-care SARS-CoV-2 salivary antigen testing with an off-the-shelf glucometer. Biosens. Bioelectron. *180*, 113111.

52. Lee, J.M., Kim, C.R., Kim, S., Min, J., Lee, M.-H., and Lee, S. (2021). Mix-and-read, one-minute SARS-CoV-2 diagnostic assay: development of PIFE-based aptasensor. Chem. Commun. *57*, 10222–10225.

53. Kraytsberg, Y., and Khrapko, K. (2005). Single-molecule PCR: an artifact-free PCR approach for the analysis of somatic mutations. Expert Rev. Mol. Diagn. *5*, 809–815.

54. Ouellet, E., Foley, J.H., Conway, E.M., and Haynes, C. (2015). Hi-Fi SELEX: a high-fidelity digital-PCR based therapeutic aptamer discovery platform. Biotechnol. Bioeng. *112*, 1506–1522.

55. Dhiman, A., Anand, A., Malhotra, A., Khan, E., Santra, V., Kumar, A., and Sharma, T.K. (2018). Rational truncation of aptamer for cross-species application to detect krait envenomation. Sci. Rep. *8*, 17795.

56. Ruscito, A., and DeRosa, M.C. (2016). Small-molecule binding aptamers: selection strategies, characterization, and applications. Front. Chem. *4*, 14.

57. Gupta, A., Anand, A., Jain, N., Goswami, S., Anantharaj, A., Patil, S., Singh, R., Kumar, A., Shrivastava, T., Bhatnagar, S., et al. (2021). A novel G-quadruplex aptamer-based spike trimeric antigen test for the detection of SARS-CoV-2. Mol. Ther. Nucleic Acids *26*, 321–332.

58. Wang, X., Gao, X., He, J., Hu, X., Li, Y., Li, X., Fan, L., and Yu, H.-Z. (2019). Systematic truncating of aptamers to create high-performance graphene oxide (GO)-based aptasensors for the multiplex detection of mycotoxins. Analyst *144*, 3826–3835.

59. Wu, C., Qavi, A.J., Hachim, A., Kavian, N., Cole, A.R., Moyle, A.B., Wagner, N.D., Sweeney-Gibbons, J., Rohrs, H.W., Gross, M.L., et al. (2021). Characterization of SARS-CoV-2 nucleocapsid protein reveals multiple functional consequences of the C-terminal domain. iScience *24*, 102681.

60. Gao, T., Gao, Y., Liu, X., Nie, Z., Sun, H., Lin, K., Peng, H., and Wang, S. (2021). Identification and functional analysis of the SARS-COV-2 nucleocapsid protein. BMC Microbiol. *21*, 58.

61. Cennamo, N., Pasquardini, L., Arcadio, F., Lunelli, L., Vanzetti, L., Carafa, V., Altucci, L., and Zeni, L. (2021). SARS-CoV-2 spike protein detection through a plasmonic D-shaped plastic optical fiber aptasensor. Talanta *233*, 122532.

62. Lewis, T., Giroux, E., Jovic, M., and Martic-Milne, S. (2021). Localized surface plasmon resonance aptasensor for selective detection of SARS-CoV-2 S1 protein. Analyst *146*, 7207–7217.

63. Aithal, S., Mishriki, S., Gupta, R., Sahu, R.P., Botos, G., Tanvir, S., Hanson, R.W., and Puri, I.K. (2022). SARS-CoV-2 detection with aptamer-functionalized gold nanoparticles. Talanta *236*, 122841.

64. Milheiro Silva, T., Akpogheneta, O., Ferreira, F., Vale-Costa, S., Medina-Lopes, M., Batista, F., Garcia, A.M., Barreto, V.M., Paulino, C., et al. (2022). Saliva molecular testing bypassing RNA extraction is suitable for monitoring and diagnosing SARS-CoV-2 infection in children. PLoS One *17*, 1–15.

65. Shrivastava, A., and Gupta, V. (2011). Methods for the determination of limit of detection and limit of quantitation of the analytical methods. Chronicles Young Sci. *2*, 21–25.

66. Cox, J., and Mann, M. (2008). MaxQuant enables high peptide identification rates, individualized p.p.b.-range mass accuracies and proteome-wide protein quantification. Nat. Biotechnol. *26*, 1367–1372.

67. Cox, J., Neuhauser, N., Michalski, A., Scheltema, R.A., Olsen, J.V., and Mann, M. (2011). Andromeda: a peptide search engine integrated into the MaxQuant environment. J. Proteome Res. *10*, 1794–1805.

68. Nagaraj, N., Kulak, N.A., Cox, J., Neuhauser, N., Mayr, K., Hoerning, O., Vorm, O., and Mann, M. (2012). System-wide perturbation analysis with nearly complete coverage of the yeast proteome by single-shot ultra HPLC runs on a bench top orbitrap. Mol. Cell. Proteomics *11*. M111.013722.

69. Cox, J., Hein, M.Y., Luber, C.A., Paron, I., Nagaraj, N., and Mann, M. (2014). Accurate proteome-wide label-free quantification by delayed normalization and maximal peptide ratio extraction, termed MaxLFQ. Mol. Cell. Proteomics *13*, 2513–2526.

70. Zuker, M. (2003). Mfold web server for nucleic acid folding and hybridization prediction. Nucleic Acids Res. *31*, 3406–3415.

71. Morozov, D., Mironov, V., Moryachkov, R.V., Shchugoreva, I.A., Artyushenko, P.V., Zamay, G.S., Kolovskaya, O.S., Zamay, T.N., Krat, A.V., Molodenskiy, D.S., et al. (2021). The role of SAXS and molecular simulations in 3D structure elucidation of a DNA aptamer against lung cancer. Mol. Ther. Nucleic Acids *25*, 316–327.

72. Maier, J.A., Martinez, C., Kasavajhala, K., Wickstrom, L., Hauser, K.E., and Simmerling, C. (2015). ff14SB: improving the accuracy of protein side chain and backbone parameters from ff99SB. J. Chem. Theor. Comput. *11*, 3696–3713.

73. Jorgensen, W.L., Chandrasekhar, J., Madura, J.D., Impey, R.W., and Klein, M.L. (1983). Comparison of simple potential functions for simulating liquid water. J. Chem. Phys. *79*, 926–935.

74. Heyer, L.J., Kruglyak, S., and Yooseph, S. (1999). Exploring expression data: identification and analysis of coexpressed genes. Genome Res. *9*, 1106–1115.

OMTN, Volume *31*

Supplemental information

Discovery of DNA aptamers targeting SARS-CoV-2 nucleocapsid protein and protein-binding epitopes for label-free COVID-19 diagnostics

Suttinee Poolsup, Emil Zaripov, Nico Hüttmann, Zoran Minic, Polina V. Artyushenko, Irina A. Shchugoreva, Felix N. Tomilin, Anna S. Kichkailo, and Maxim V. Berezovski

SARS-CoV-2 Protein expression

DH5α strain with a plasmid encoding for N protein was received from Rick Tarleton (Addgene, Watertown, MA). An LB agar plate containing 100 µg/mL kanamycin (Sigma-Aldrich, St. Louis, MO) was inoculated with the culture and incubated at 37°C for 24 hours. A single colony was picked and inoculated into 5 mL LB with 100 µg/mL kanamycin, which was incubated at 300 rpm and 37°C overnight. The plasmid was extracted using a miniprep kit (Qiagen, Germantown, MD), and 10 ng of the plasmid was used to transform into competent cells (DE3 strain) (Thermo Fisher Scientific, Waltham, MA) according to the manufacturer's protocol. The transformed competent cells were cultured in the same conditions as DH5α and were frozen in 50% glycerol/LB. The frozen cells were inoculated into 200 mL LB with 100 µg/mL kanamycin. The culture was incubated at 37°C on a magnetic stir plate at 300 rpm until a OD600 reached 1. The culture was then supplied with 1mM IPTG (Sigma-Aldrich, St. Louis, MO) and further incubated for 3 hours. Cells were spun down at 4,000 g for 30 minutes and resuspended in T300 buffer (30mM triethanolamine, 300 mM NaCl, pH 7.8). 1mg/mL of lysozyme and 1mM PMSF (Sigma-Aldrich, St. Louis, MO) were added before sonication for 30 minutes on ice. The lysate was centrifugated at 21,000 g for 40 minutes, and pellets were resuspended in T300 supplied with 8 M urea (Sigma-Aldrich, St. Louis, MO). The solubilized pellets were then filtered through a 0.2-µm nylon membrane. The C 10/10 chromatography column (Cytiva, Marlborough, MA) was packed with 7.85 mL of Ni-NTA agarose beads (Thermo Fisher Scientific, Waltham, MA). Packed media was equilibrated with T300 buffer containing 8 M urea for 4 column volumes. 10 mL of solubilized sample was injected into the column, which was then washed with 4 more column volumes of the same buffer containing urea. The buffer was slowly replaced with T300 by applying gradient for 8 column volumes to gradually remove urea and refold protein on the column. Column was then

washed with T300 with 30 mM imidazole for 4 column volumes. The protein was then eluted with 8 column volumes of elution buffer (T300 and 300mM imidazole) and concentrated on a 10 kDa filter (Sigma-Aldrich, St. Louis, MO). All chromatography steps were performed using a flow rate of 3 mL/min. In the next step, gel filtration chromatography was performed using a XK 16/70 column (Cytiva, Marlborough, MA) which was packed with 140 mL of Sephacryl S-100 HR resin (Cytiva, Marlborough, MA). The column was washed with 2 volumes of gel filtration buffer (50 mM HEPES, 50 mM NaCl and 10 mM $MgCl_2$, pH 7.5). 1 mL of concentrated sample was injected and separated at a flow rate of 0.5 mL/min. A280 was monitored during the run to collect fractions of N protein. The collected protein was then concentrated on a 10 kDa filter to the final volume of 1 mL and stored at -20 °C until further use.

Table S1. Full-length sequences (80 nt) of all selected aptamer candidates were obtained from NGS data enrichment. The primer-binding regions are written in bold.

Aptamer	Aptamer sequence
NSP1	**CTCCTCTGACTGTAACCACG**GCGCAAGCCGGGGTGTACGTGTTATACGTGCGTGTATCGA**GCATAGGTAGTCCAGAAGCC**
NSP2	**CTCCTCTGACTGTAACCACG**TATTGCGTTCCAGTCCCTATGACCAACGTCACAATAAGTC**GCATAGGTAGTCCAGAAGCC**
NSP3	**CTCCTCTGACTGTAACCACG**CAGCGTCACGTGTTGTTCCCCATTGTACTGATTCGTCGTG**GCATAGGTAGTCCAGAAGCC**
NSP4	**CTCCTCTGACTGTAACCACG**CGTTGAGCGTGTTGTCCCTACATGCAATAGACCCTCCTTC**GCATAGGTAGTCCAGAAGCC**
NSP5	**CTCCTCTGACTGTAACCACG**ATTCATGCGCCAATAGTGGTTTGGAAATGTCTCCCCATAC**GCATAGGTAGTCCAGAAGCC**
NSP6	**CTCCTCTGACTGTAACCACG**GCATAGGTAGTCAAGAAGCCATCTCCTATGACTGTAACCACG**GCATAGGTAGTCCAGAAGCC**
NSP7	**CTCCTCTGACTGTAACCACG**TGCTAGATTACGTAACTGGTTGCTACGGGTTTCATCCTCG**GCATAGGTAGTCCAGAAGCC**
NSP8	**CTCCTCTGACTGTAACCACG**AGGATTGCGCACATACGGTTGCTCACCATTCTACTGTCCT**GCATAGGTAGTCCAGAAGCC**
NSP9	**CTCCTCTGACTGTAACCACG**CTTATGCACCAGGGGCCCGGTTCGGGCAGGCGCAAACACG**GCATAGGTAGTCCAGAAGCC**
NSP10	**CTCCTCTGACTGTAACCACG**GCATAGGTAGTCAAGAAGCCATCTCATCTGACTGTAACCACG**GCATAGGTAGTCCAGAAGCC**
NSP11	**CTCCTCTGACTGTAACCACG**GCGCACCCTGCGACGTCCTCATGCCAGGCTTCAGCCTCAA**GCATAGGTAGTCCAGAAGCC**
NSP12	**CTCCTCTGACTGTAACCACG**TGGTTCGCCCAGTTCCGTTCAATACACTCAACCCTGCACG**GCATAGGTAGTCCAGAAGCC**
NSP13	**CTCCTCTGACTGTAACCACG**TATTGCGTTCCATTCCCTATGACCAACGTCACAATAAGTC**GCATAGGTAGTCCAGAAGCC**
NSP14	**CTCCTCTGACTGTAACCACG**GGCTTGGTTTCGGGATCTGCACCCCTGAAACATTTCTCAG**GCATAGGTAGTCCAGAAGCC**
NSP15	**CTCCTCTGACTGTAACCACG**CTCGTCCAGCGGCTGCGTGTTTCACCGTGCATTGTTGATA**GCATAGGTAGTCCAGAAGCC**
NSP16	**CTCCTCTGACTGTAACCACG**TTCAAGAGCGGTTCGTAATGTTAATTGACTGATCCCTACC**GCATAGGTAGTCCAGAAGCC**

Table S2. The truncated aptamer sequences and their binding parameters interacting with His-tagged N protein immobilized on the Ni-NTA biosensors.

Aptamer	Aptamer sequence	K_D (nM)	k_a (1/Ms)	k_d (1/s)
tNSP5 (46nt)	ATTCATGCGCCAATAGTGGTTTGGAA ATGTCTCCCCATACGCATAG	12.6±2.56	$8.25x10^5$	$1.07x10^{-2}$
tNSP9 (60nt)	TGTAACCACGTGCTAGATTACGTAAC TGGTTGCTACGGGTTTCATCCTCGGCA TAGGTA	22.2±1.87	$4.77x10^5$	$1.06x10^{-2}$
tNSP10 (47nt)	CTTATGCACCAGGGGCCCGGTTCGGG CAGGCGCAAACACGGCATAGG	8.60±1.20	$7.44x10^5$	$6.39x10^{-3}$
tNSP12 (60nt)	TGTAACCACGTGGTTCGCCCAGTTCCG TTCAATACACTCAACCCTGCACGGCA TAGGTA	6.05±2.41	$6.14x10^5$	$3.99x10^{-3}$
A48 (58 nt)	GCTGGATGTCGCTTACGACAATATTCC TTAGGGGCACCGCTACATTGACACAT CCAGC	6.46±1.04	$7.82x10^5$	$5.05x10^{-3}$
A58 (58 nt)	GCTGGATGTCACCGGATTGTCGGACA TCGGATTGTCTGAGTCATATGACACAT CCAGC	3.09±0.95	$3.58x10^5$	$1.08 x10^{-3}$

Table S3. Hydrogen bonds in the N protein-tNSP3 complexes. The number in parentheses indicates the conformer number. Amino acids of AA10 peptide are in bold.

N protein/NSP3 (1)		N protein/NSP3 (2)		N protein/NSP3 (1)		N protein/NSP3 (2)	
**Binding site AA21**		**Binding site AA21**		**Binding site AA10**		**Binding site AA10**	
Residue	Nucleotide	Residue	Nucleotide	Residue	Nucleotide	Residue	Nucleotide
GLN 9	G 35	GLN 9	G 35	LYS 338	C 4	ASN 126	T 23
ARG 10	G 35	ARG 10	G 35	LYS 361	C 37	LYS 127	T 23
ARG 14	C 34	ASN 29	C 1	GLU 367	G 30	ARG 276	T 23
ASN 29	T 6	**ASN 77**	T 32	**ARG 385**	T 17	ARG 293	C 19
ASN 29	A 2	**SER 79**	T 23	**GLN 384**	G 15	LYS 338	C 4
GLU 31	C 1	THR 14	C 21	**GLN 384**	C 18	LYS 361	C 37
GLU 31	A 2	LYS 143	C 19	GLN 386	T 14	GLU 367	G 30
SER 37	C 1	LYS 143	C 20	LYS 387	T 16	LYS 373	T 29
**ASN 77**	T 32	GLN 160	G 25	LYS 387	T 14	LYS 375	T 33
**SER 79**	A 22	ARG 189	A 27	GLN 408	C 4	**GLN 384**	G 15
**SER 79**	T 23	SER 193	T 29	GLN 408	T 6	**GLN 384**	C 18
LYS 143	C 20	LYS 237	G 3			**GLN 384**	C 19
GLN 160	G 25					**ARG 385**	T 17
GLN 163	T 24					**ARG 385**	C 18
SER 193	T 29					GLN 386	T 14
SER 201	T 29					GLN 386	T 16
ARG 203	G 30					LYS 387	T 16
ARG 203	T 29					GLN 408	C 4
LYS 237	G 3						
LYS 256	A 2						

A

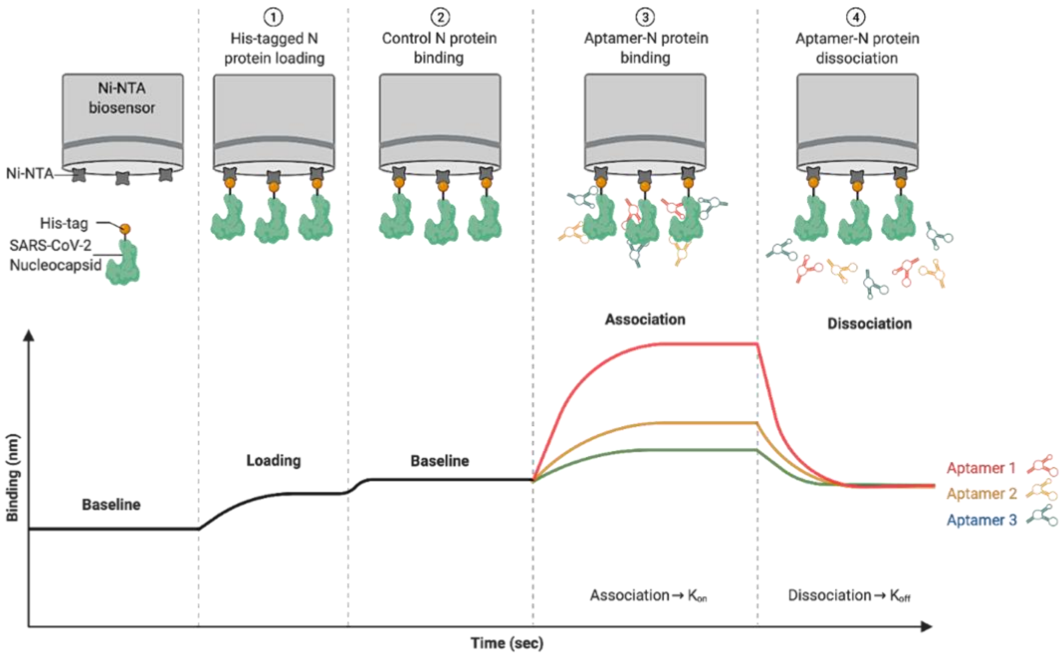

B

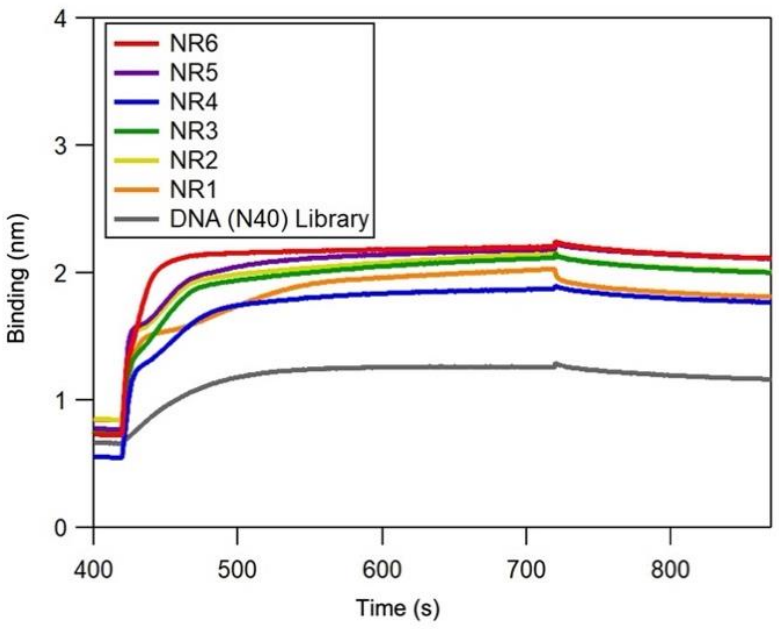

Figure S1. **BLI affinity tests.** (A) Schematic BLI workflow of aptamers binding to N protein. (B) BLI sensorgrams showing the association and dissociation curves of the six enriched pools obtained from the aptamer selection.

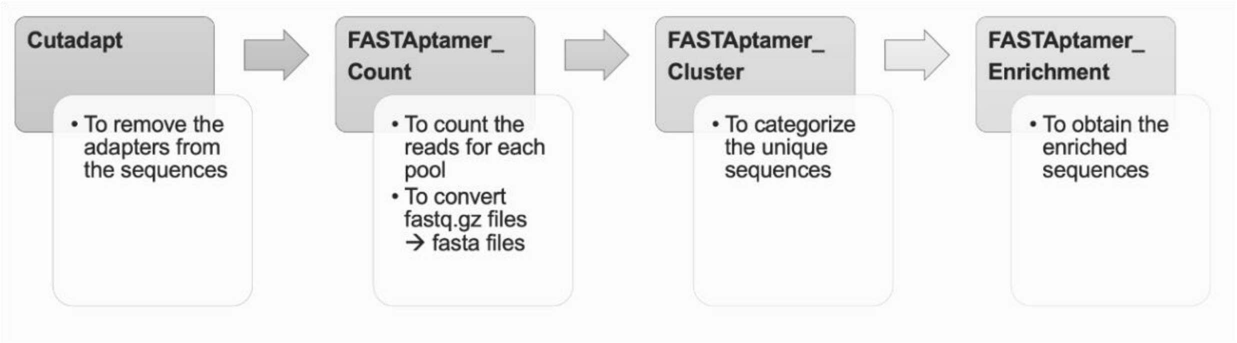

Figure S2. Schematic NGS data analysis. The data processing workflow to retrieve the aptamer sequences from NGS raw data.[36]

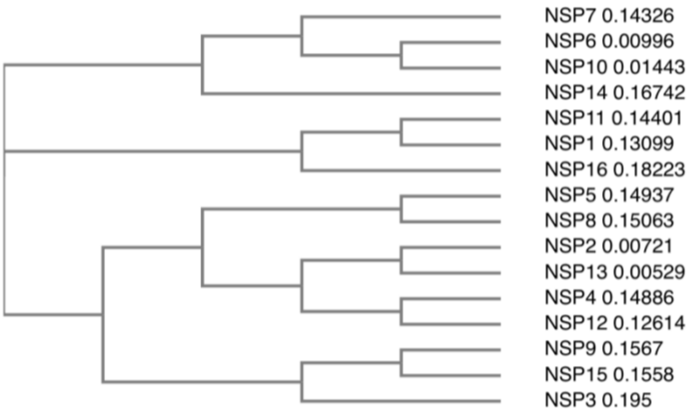

Figure S3. **Phylogenetic tree of DNA aptamers.** The diagram obtained from Clustal Omega represents the three main groups of sixteen enriched aptamer sequences.

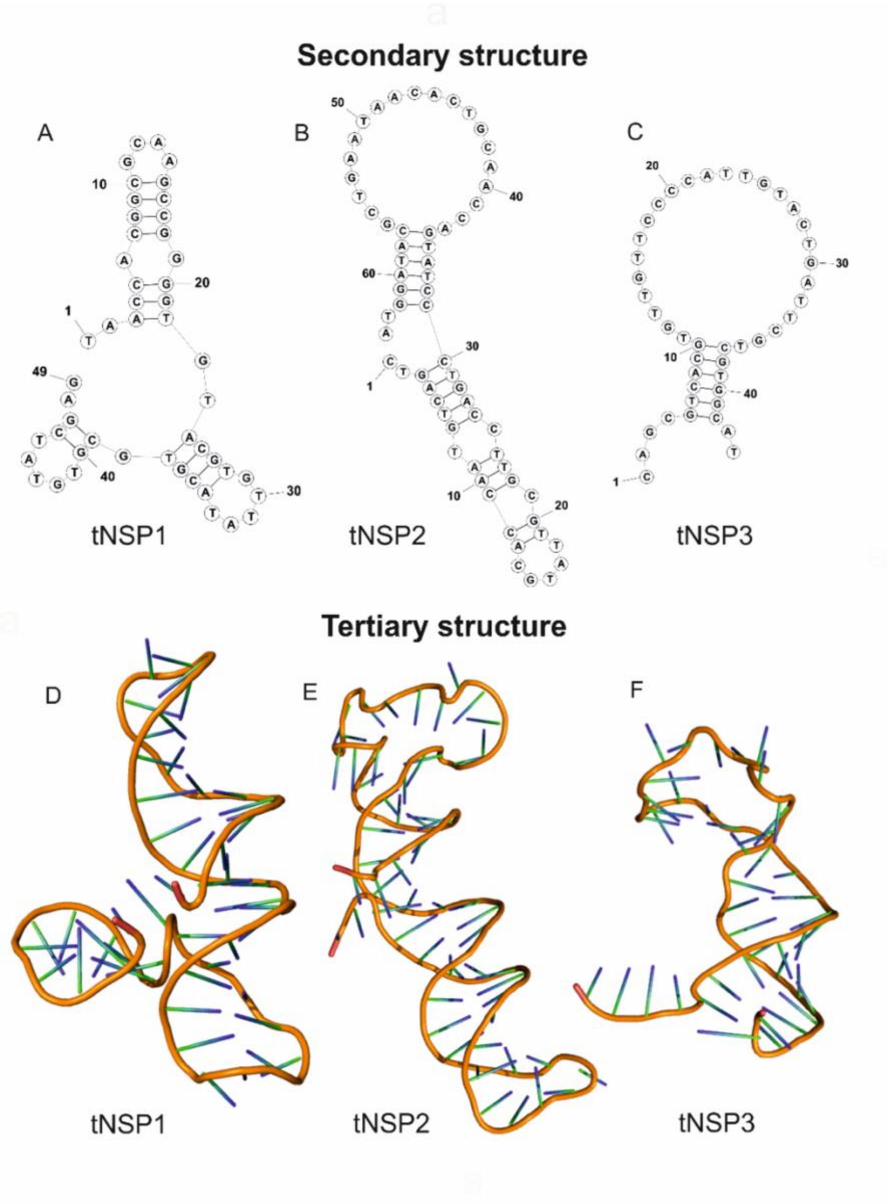

Figure S4. The structural prediction of truncated aptamers. Secondary structures (A-C) of tNSP1, tNSP2, and tNSP3 aptamers predicted by the mFold web server.[68] Tertiary structures (D-E) obtained from MD simulations (200 ns).

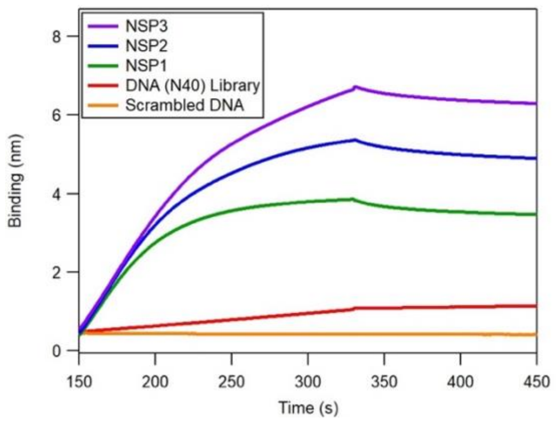

Figure S5. **Binding evaluation on BLI.** Binding affinity tests of NSP1, NSP2, and NSP3 aptamers with the N protein. The initial DNA N40 library and scrambled DNA were used as controls.

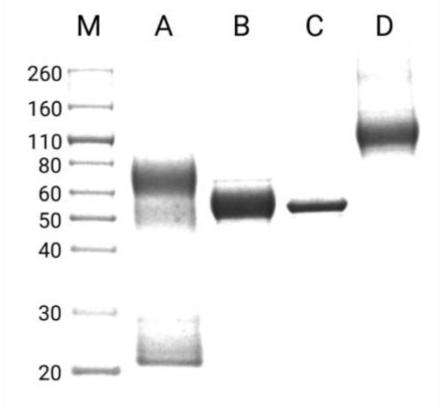

Figure S6. SDS-PAGE gel image with four proteins. M – molecular marker; A – nucleocapsid of MERS-CoV; B – nucleocapsid of SARS-CoV-2 obtained from ACROBiosystems; C – nucleocapsid of SARS-CoV-2 expressed in BL-21 *E. coli*. D. S1 subunit of the spike protein of SARS-CoV-2 obtained from ACROBiosystems.

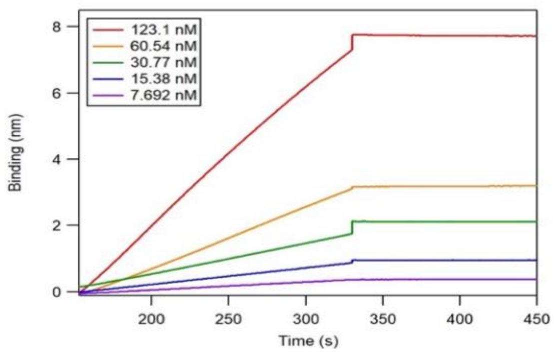

Figure S7. **BLI sensorgrams of tNSP3 aptamer.** The binding assay determining K_D of tNSP3 aptamer binding to our in-house expressed SARS-CoV-2 nucleocapsid protein at 7.69, 15.38, 30.76, 61.52, and 123.04 nM.
